# Immuno-transcriptomic profiling of extracranial pediatric solid malignancies

**DOI:** 10.1016/j.celrep.2021.110047

**Published:** 2021-11-23

**Authors:** Andrew S. Brohl, Sivasish Sindiri, Jun S. Wei, David Milewski, Hsien-Chao Chou, Young K. Song, Xinyu Wen, Jeetendra Kumar, Hue V. Reardon, Uma S. Mudunuri, Jack R. Collins, Sushma Nagaraj, Vineela Gangalapudi, Manoj Tyagi, Yuelin J. Zhu, Katherine E. Masih, Marielle E. Yohe, Jack F. Shern, Yue Qi, Udayan Guha, Daniel Catchpoole, Rimas J. Orentas, Igor B. Kuznetsov, Nicolas J. Llosa, John A. Ligon, Brian K. Turpin, Daniel G. Leino, Shintaro Iwata, Irene L. Andrulis, Jay S. Wunder, Silvia R.C. Toledo, Paul S. Meltzer, Ching Lau, Beverly A. Teicher, Heather Magnan, Marc Ladanyi, Javed Khan

**Affiliations:** 1 Sarcoma Department, H. Lee Moffitt Cancer Center and Research Institute, Tampa, FL 33612, USA; 2 Genetics Branch, CCR, NCI, NIH, Bethesda, MD 20892, USA; 3 Advanced Biomedical Computational Science, Leidos Biomedical Research Inc., NCI Campus at Frederick, Frederick, MD 21702, USA; 4 Laboratory of Pathology, CCR, NCI, NIH, Bethesda, MD 20892, USA; 5 Cancer Research UK Cambridge Institute, University of Cambridge, Cambridge, UK; 6 Pediatric Oncology Branch, CCR, NCI, NIH, Bethesda, MD 20892, USA; 7 Thoracic and GI Malignancies Branch, CCR, NCI, NIH, Bethesda, MD 20892, USA; 8 The Tumour Bank, Children’s Cancer Research Unit, Kids Research Institute, The Children’s Hospital at Westmead, Westmead, NSW, Australia; 9 Ben Towne Center for Childhood Cancer Research, Seattle Children’s Research Institute, Seattle, WA 98101, USA; 10 Department of Pediatrics, University of Washington School of Medicine, Seattle, WA 98101, USA; 11 Cancer Research Center and Department of Epidemiology and Biostatistics, School of Public Health, University at Albany, Rensselaer, NY 12144, USA; 12 Pediatric Oncology, John Hopkins University School of Medicine, Baltimore, MD 21218, USA; 13 Division of Oncology, Cincinnati Children’s Hospital, 3333 Burnet Avenue, Cincinnati, OH 45229-3026, USA; 14 Chiba Cancer Center, Chiba, Japan; 15 Lunenfelf-Tanenbaum Research Institute, Sinai Health System; Department of Molecular Genetics, University of Toronto, Toronto, ON, Canada; 16 University of Toronto Musculoskeletal Oncology Unit, Sinai Health System; Department of Surgery, University of Toronto, Toronto, ON, Canada; 17 Support Group for Children and Adolescents with Cancer (GRAACC), Pediatric Oncology Institute (IOP), Universidade Federal de Sao Paulo, Sao Paulo, Brail; 18 The Jackson Laboratory, Farmington, CT 06032, USA; 19 Molecular Pharmacology Branch, DCTD, NCI, NIH, Bethesda, MD 20892, USA; 20 Department of Pediatrics, Memorial Sloan Kettering Cancer Center, New York, NY, USA; 21 Department of Pathology, Memorial Sloan Kettering Cancer Center, New York, NY, USA; 22 These authors contributed equally; 23 Lead contact

## Abstract

We perform an immunogenomics analysis utilizing whole-transcriptome sequencing of 657 pediatric extra-cranial solid cancer samples representing 14 diagnoses, and additionally utilize transcriptomes of 131 pediatric cancer cell lines and 147 normal tissue samples for comparison. We describe patterns of infiltrating immune cells, T cell receptor (TCR) clonal expansion, and translationally relevant immune checkpoints. We find that tumor-infiltrating lymphocytes and TCR counts vary widely across cancer types and within each diagnosis, and notably are significantly predictive of survival in osteosarcoma patients. We identify potential cancer-specific immunotherapeutic targets for adoptive cell therapies including cell-surface proteins, tumor germline antigens, and lineage-specific transcription factors. Using an orthogonal immunopeptidomics approach, we find several potential immunotherapeutic targets in osteosarcoma and Ewing sarcoma and validated PRAME as a bona fide multi-pediatric cancer target. Importantly, this work provides a critical framework for immune targeting of extracranial solid tumors using parallel immuno-transcriptomic and -peptidomic approaches.

## In brief

Brohl et al. perform immunogenomics analysis of a cohort of 788 pediatric extracranial solid tumors and cell lines, representing 14 diagnoses, utilizing RNA sequencing. They broadly describe prognostically relevant immunophenotypes and provide proof of concept of a transcriptomics-informed adoptive cellular therapy approach, validating *PRAME* as a multi-pediatric cancer immunotherapy target.

## Graphical Abstract

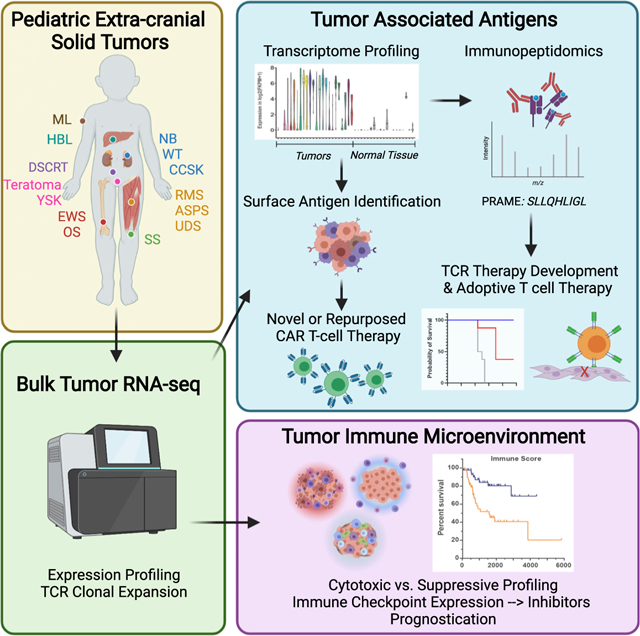

## INTRODUCTION

Pediatric malignancies remain the leading cause of disease-related death in children in the United States. While advances in multidisciplinary treatment in the later parts of the 20^th^ century resulted in significant improvements in survival from pediatric cancer, further progress over the last several decades has been modest (Howlader et al., 2014). For most pediatric malignancies, especially solid tumors, survival remains poor for those with relapsed or advanced disease.

Rarity and heterogeneity make pediatric cancers challenging to study and treat. Increasingly, there is a desire to apply high-throughput analytical techniques to better characterize tumor’s molecular composition for precision therapeutics. Recently, two large next-generation sequencing studies have helped to provide the landscape of the most common mutational drivers across a range of pediatric cancers (Gröbner et al., 2018; Ma et al., 2018). While these studies form a foundation for our molecular understanding of these malignancies, further work is needed to enable translation to the clinic.

In recent years, immunotherapy has revolutionized the treatment approach for an increasing number of adult cancer types. These clinical breakthroughs have led to a heightened interest in understanding the molecular basis for immunotherapy response. To further our understanding of the immunogenomic landscape in pediatric malignancies, we performed an analysis of whole-transcriptome sequencing of 657 extracranial solid tumor specimens, representing 14 major cancer diagnoses. A comprehensive analysis of the immunogenomic features of these tumors was performed including tumor microenvironment and immune infiltration pattern with quantification, expression pattern of immune checkpoint genes, tumor-specific genes, and intratumoral T cell receptor (TCR) repertoire. We further provided orthogonal confirmation of transcriptomics findings of immunotherapeutic targets by evaluating the immunopeptidome of osteosarcoma and Ewing sarcoma and demonstrated proof-of-concept *in vitro* and *in vivo* cytotoxicity using a transcriptomics-informed adoptive cell therapy (ACT) approach.

## RESULTS

Whole-transcriptome sequencing analysis was performed on 657 tumor samples from 623 pediatric or young adult patients diagnosed with an extra-cranial solid malignancy. In parallel, RNA sequencing was performed on 131 commonly used cancer cell lines and 147 normal tissues representing 21 organs (Table 1; Table S1A). Quality metrics of the sequencing data are summarized in Table S1B and S1C. Fourteen different diagnoses, including sub-categories of neuroblastoma (NB; *MYCN* amplified and not amplified) and rhabdomyosarcoma (RMS; fusion positive and negative) were represented (Table 1; Table S1A). Mirroring the relative disease incidence of extracranial pediatric solid malignancies seen at our sites, the majority of our cohort comprised one of four diagnoses: neuroblastoma, rhabdomyosarcoma, Ewing sarcoma, and osteosarcoma.

### Tumor immune microenvironment

**Figure 1.**
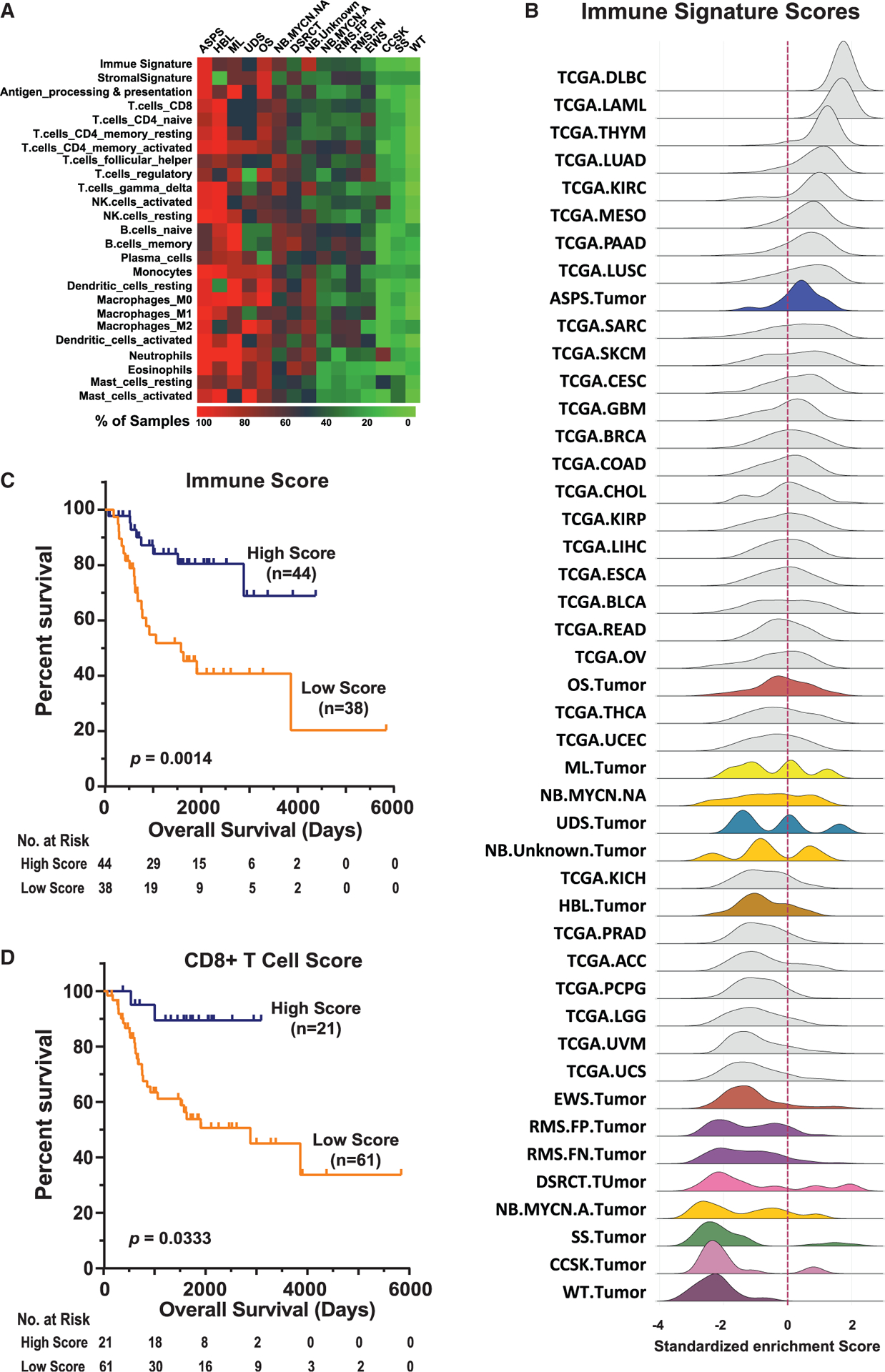
Tumor immune microenvironment of pediatric solid tumors (A) Global pattern of enrichment of various immune signatures across cancer types. Tumor types with a sample size of >5 are shown. The heatmap corresponds to the percentage of tumors with a positive enrichment score for the immune cell subtype by ssGSEA. (B) Distribution of immune signature enrichment scores across cancer types included in this study (colored) as compared to adult tumor samples in the TCGA project (gray). (C and D) Kaplan-Meier (KM) plots of overall survival demonstrate that patients with tumors of high immune score (C) or high CD8^+^ T cell score (D) are significantly associated with a favorable prognosis in the osteosarcoma cohort where outcome data are available.

To characterize the immune microenvironment in pediatric solid tumors, we performed tumor profiling of infiltrating immune cells using predefined immune gene sets, including CIBERSORT, immune, and stromal signatures where enrichment scores for immune cell subtypes as well as a described overall “immune signature” in each tumor sample were calculated (Newman et al., 2015; Yoshihara et al., 2013). First, we assessed enrichment scores for immune cell types and gene signatures in each tumor in the context of its tumor type (Figure 1A) and observed diverse enrichment of immune signatures among different types of tumors. There was a strong correlation between immune signature score and enrichment for all immune cell subtypes (Figure 1A). Interestingly, there was a notable clustering of fusion-driven malignancies such as Ewing sarcoma (EWS), fusion-positive RMS, synovial sarcoma (SS), clear cell sarcoma of kidney (CCSK), and desmoplastic small round cell tumor (DSRCT) as having low median infiltrating immune cell enrichments relative to other tumor types in the analysis. A notable exception to this pattern is alveolar soft part sarcoma (ASPS), which had the highest median immune signature score among tumor types in our cohort despite being a fusion driven malignancy. On the other hand, tumor types with the highest relative median immune cell enrichment include diseases reported to have “complex” genomes including osteosarcoma (OS), melanoma (ML), and undifferentiated sarcoma (UDS) (Figure 1A).

To compare pediatric tumors with adult malignancies, we performed a combined analysis of our cohort with publicly available data from The Cancer Genome Atlas (TCGA). For the overall immune signature scores, all pediatric tumor subtypes displayed modest or below median immune signature scores with corresponding modest CD8^+^ T cell enrichment relative to adult tumor counterparts, with the notable exception of ASPS, which was the only pediatric tumor to fall within the top quartile of median enrichment (Figure 1B; Figure S1). Remarkably, ASPS displayed the highest levels of human leukocyte antigen A (HLA-A) expression across all tumor types evaluated, including TCGA adult tumors (Figure S1). These results are notable in light of the recent clinical observation that ASPS is one of the most clinically responsive tumor types to checkpoint inhibitor therapy (Wilky et al., 2019), and the association between expression of major histocompatibility complex (MHC) molecules and efficacy of checkpoint inhibitory therapies (Yuasa et al., 2017).

We next sought to evaluate the potential impact of tumor immune microenvironment on prognosis. For this analysis, we utilized the osteosarcoma patient cohort as outcomes data were available for this subgroup in our cohort (Table S1D). Of note, all tumor samples used for this analysis were taken from the primary tumor prior to any chemotherapy. Remarkably, similar to our previously reported association between high immune cell infiltrate enrichment and favorable prognosis in neuroblastoma (Wei et al., 2018), we found that increased expression of immune cell markers in the tumor microenvironment of these pre-treatment osteosarcoma tumors were significantly associated with a more favorable outcome (Figures 1C and 1D). The associations remain highly significant in cox-regression analysis to account for metastatic status, indicating that the presence of CD8^+^ T cells in pre-treatment primary tumors is an independent predictor for patient outcome (Figure S2).

### Immune checkpoint expression

**Figure 2.**
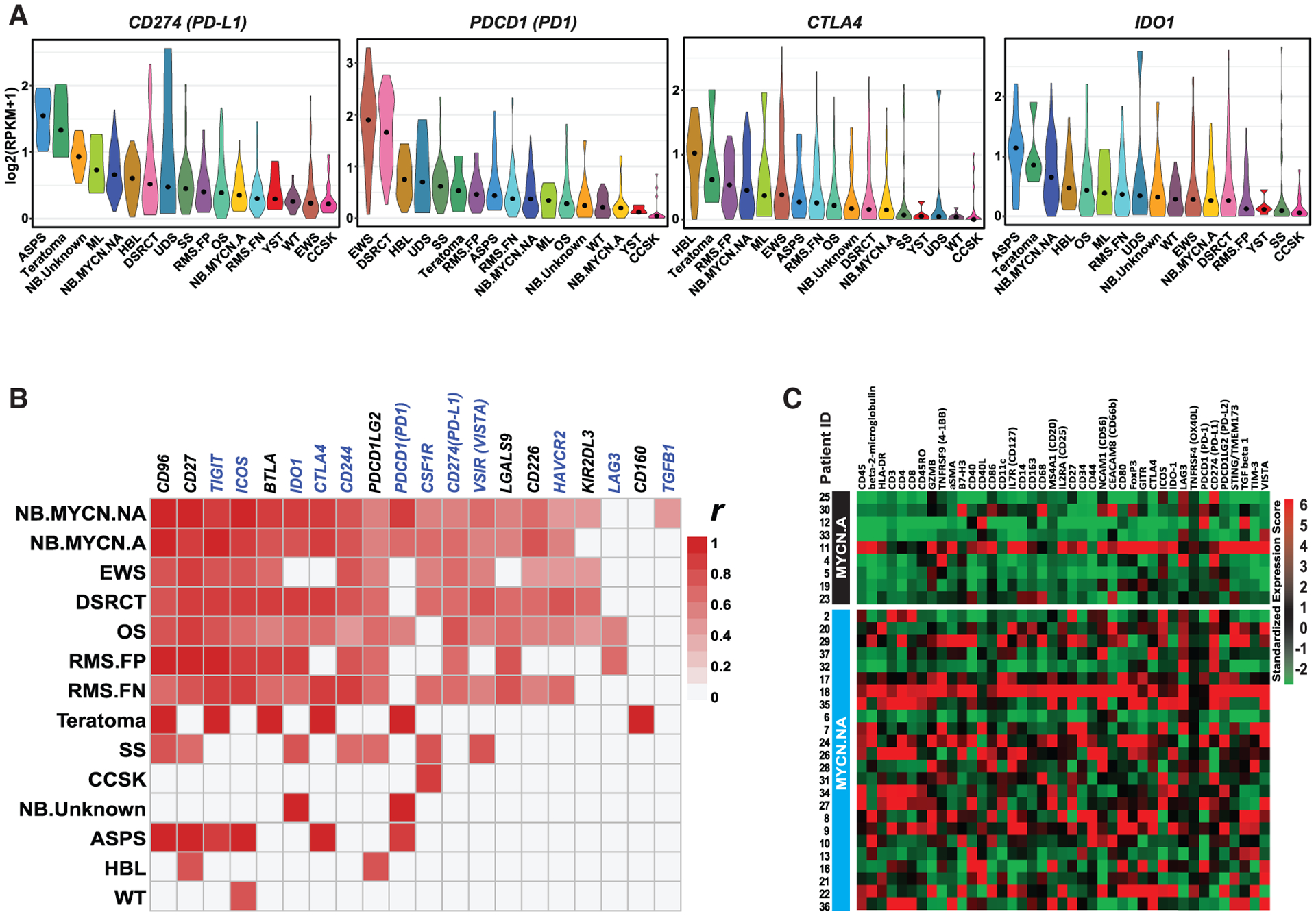
Immune checkpoint expression in pediatric solid tumors (A) Expression of selected immune checkpoint genes across tumor types. Dots represent the median expression for each cancer type. (B) Correlation of immunomodulatory gene expression and CD8^+^ T cell infiltrate. Fill indicates significant association (Spearman rank correlation >0.3; adjusted p < 0.05) within that cancer type. Highlighted genes in the blue font represent targets of antibody therapies approved by FDA or currently in clinical trial. (C) Protein expression of an immune gene panel on an independent neuroblastoma tissue array (Wei et al., 2018) using a multiplex protein detection assay reveal consistent findings of differential expression of immune cell markers between *MYCN*-amplified (*MYCN.A*) and *MYCN*-not amplified (*MYCN.NA*) tumors. The scale bar represents *Z*-scored standardized protein-expression level.

Given the rapid development of checkpoint inhibitor therapy across many tumor types, expression of immunoinhibitory molecules (e.g., CD274 or PD-L1) is of translational interest. Similar to the immune cell infiltrate enrichment patterns, we observed heterogeneity in the expression of immune checkpoint genes both within and across tumor types (Figure 2A; Figure S3). Consistent with its sensitivity to immune checkpoint blockade, ASPS demonstrated the highest median expression of *PD-L1* suggesting a dependency on this immune checkpoint for immune evasion (Figure 2A). To further evaluate for immune checkpoints that might be the most clinically relevant in our cohort, we performed a correlation analysis between the expression of individual immune checkpoint genes and CD8^+^ T cell infiltrate enrichment within cancer types to look for the co-occurrence of these two features. Interestingly, we observed two patterns of immune checkpoint expression correlates to CD8^+^ T cell infiltrate enrichment (Figure 2B). First was a cluster of cancer types including NB, EWS, DSRCT, OS, and RMS in which the majority of checkpoint genes evaluated are significantly correlated with tumor-infiltrating CD8^+^ T cells (Spearman rank correlation >0.3; adjusted p < 0.05). On the contrary, in the remaining tumor types including ASPS, a much narrower set of immune modulating genes are significantly associated with tumor-infiltrating CD8^+^ T cells (Figure 2B).

Expanding on our prior observations in neuroblastoma (Wei et al., 2018), we noted appreciable differences in TIL enrichment pattern (Figure 1A) and expressional levels of immune checkpoints between *MYCN*-not amplified (*MYCN.NA*) and MYCN-amplified (*MYCN.A*) tumors (Figure 2A). To validate these findings at the protein level, we performed immunoprofiling using multiplex NanoString digital spatial profiling technology on a neuroblastoma tissue array containing 33 independent neuroblastoma tumors of which 9 were *MYCN.A* and 24 were *MYCN.NA* (Wei et al., 2018). Consistent with the RNA sequencing (RNA-seq) data, the protein-expression levels of TIL infiltrate markers and immune checkpoints are generally elevated in *MYNC.NA* tumors compared to *MYCN.A* tumors (Figure 2C).

Despite disappointing early trials with immune checkpoint inhibitors, osteosarcoma has been proposed as a potentially immune responsive tumor due to favorable immunogenomic characteristics, including relatively high immune cell infiltration when compared to other pediatric cancer types. We then selected osteosarcoma to further validate the presence of immune cell types and immune checkpoint expression as predicted by RNA-seq. Multiplexed immunohistochemistry was performed for a panel of immune markers in an independent cohort of 25 osteosarcoma tumors. Consistent with the RNA-seq prediction and the previous findings in a subset of TARGET osteosarcoma cohort (Wunder et al., 2020), there were robust infiltrating T cells (markers CD3, CD8) and M2 macrophages (CD163), with moderate PD-L1 expression in these osteosarcoma samples (Figure S4). On the other hand, relative to other pediatric solid tumor types, we observed osteosarcoma to exhibit pronounced expression of additional immunosuppressive molecules particularly *TGFB1* and *CSF1R*, where median expression was the highest among all tumor types in the study cohort (Figure S3).

### T cell receptor repertoire

**Figure 3.**
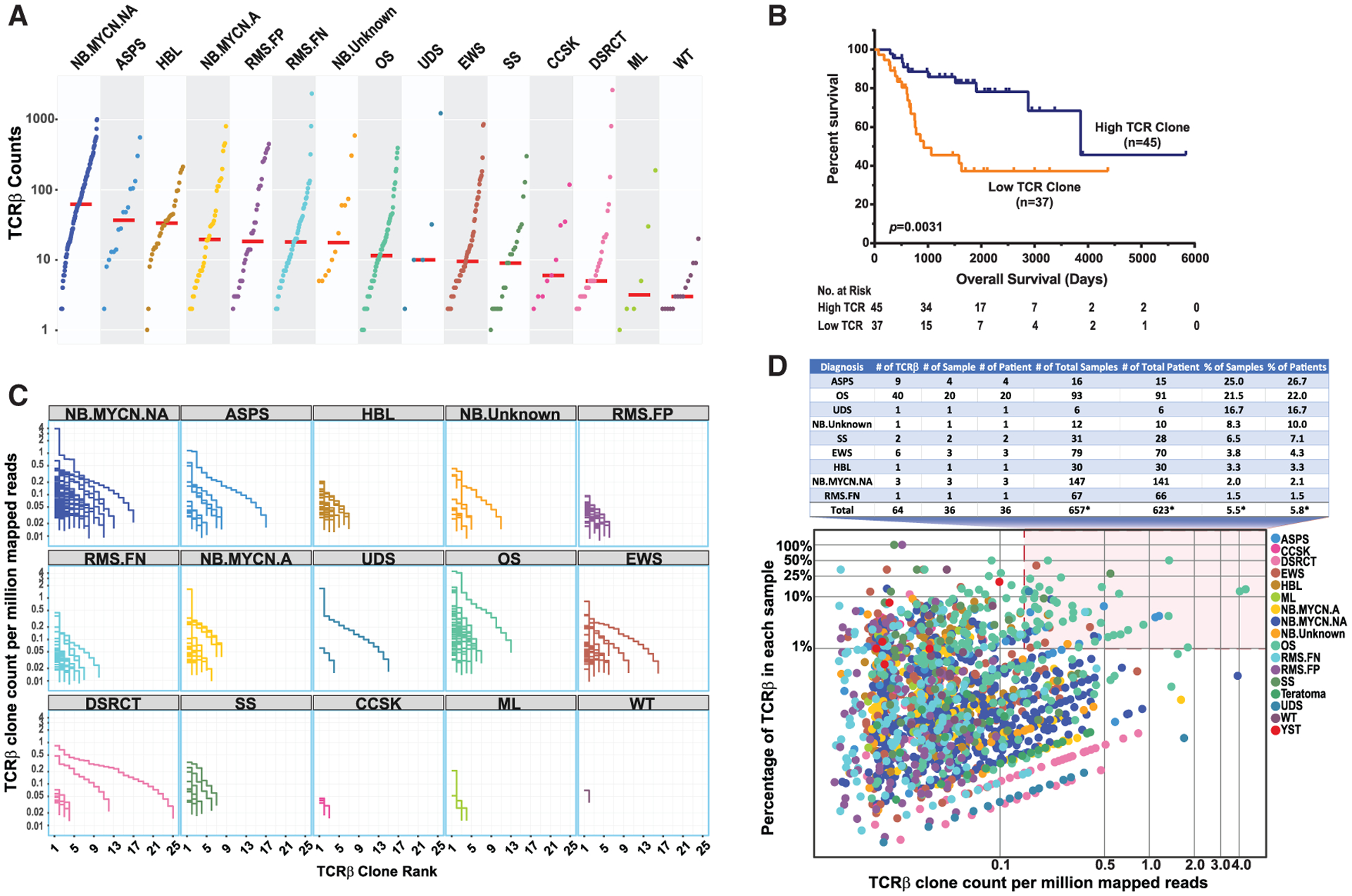
Intra-tumoral T cell receptor β (TCR-β) repertoire identified using RNA-seq data in pediatric solid tumors (A) Number of unique complementary-determining region 3 (CDR3) detected in each tumor. Red bars represent median for each cancer type. (B) Kaplan-Meier analysis of available outcome data in the osteosarcoma cohort demonstrates that patients with a high TCR-β count are significantly associated with favorable outcome (p < 0.01). (C) In order to investigate T cell clone expansion in individual tumors, TCR-β clones are ranked by their abundance on the x axis and the normalized clone count is plotted on the y axis. Each line represents all TCR-β clones detected in a single tumor and clearly shows evidence of high clonal expansion of some TCRs. (D) Clonal expansion of TCR-βs. Each dot represents a TCR-β clone in a tumor sample. The highlighted region depicts expanded TCR-β clones as evidenced by high normalized clone count (>99^th^ percentile) and high relative contribution to the total intra-tumoral TCR-β count (>1%). The accompanying table details the percentage of tumors with ≥1 clonally expanded TCR-β. *Total tumor/patient count and calculated percentages include all patients in the study cohort.

To further characterize the immunologic landscape of pediatric solid tumors, we performed an analysis of the T cell receptor (TCR) repertoire utilizing a computational method for *de novo* assembly of hypervariable region sequences from complementary-determining region 3 (CDR3) (Li et al., 2016). In total, we identified 35,897 unique T cell receptor beta-chain (TCR-β)-CDR3 sequences across our tumor cohort (Figure 3A; Table S2). Of note, *MYCN*-not-amplified NB and ASPS had the highest median TCR-β counts among the tumor types studied, in keeping with their responsiveness to immune therapy. The CDR3 sequences had lengths ranging from 6 to 25 amino acids with a median length of 14 (Figure S5A). The sequence patterns for the most frequent 14-amino-acid CDR3 sequence were very similar to that previously reported across a large set of adult cancers (TCGA) as well as in the peripheral blood of healthy donors (Figure S5) (Li et al., 2016; Warren et al., 2011).

We next compared TCR sequences identified within our tumor cohort to those identified across the TCGA database (Li et al., 2016), to those identified in normal tissues from our study cohort, and to those reported in two healthy donor population databases (Shugay et al., 2018; Warren et al., 2011) (Table S2; Figure S5F). Of the 35,897 total TCRs identified across our tumor cohort, 29,381 (84.7%) were unique to a single tumor and not present in the comparator databases, 5,327 (15.3%) overlapped with one of the healthy adult databases or normal tissues. Of note, out of 194 shared TCR sequences in 2 or more tumors, 165, represented by unique protein sequences, were shared in 2 or more patients within our cohort and not present in the comparator healthy population databases, which may represent tumor targeting TCRs (Table S2; Figure S5F).

We evaluated the potential impact of intra-tumoral TCR burden (measured as TCR counts) on outcome in the OS cohort where clinical data were available. Similar to our analysis of immune score and CD8^+^ T cell infiltrate enrichment, we found that patients with a high TCR clone burden in these pre-treatment samples were significantly associated with a more favorable prognosis (Figure 3B), which was independent of the metastatic status of the patient (Figure S2).

To evaluate for possible clonal expansion of TCRs, we determined the abundance of each CDR3 clone (counts/million) as well as the relative contribution of each TCR clone to the total TCR counts found in each tumor. Indeed, we noted several tumors that had evidence of high intra-tumoral TCR burden with varying degrees of TCR diversity (Figure 3C). When quantifying expanded TCR clones as defined by both high expression (>99^th^ percentile) and high relative contribution to total intra-tumoral TCR count in a given tumor (>1%), we observed 36/623 (5.8%) of patients (range 0%–26.7% per cancer type) having a tumor that met our criteria for intra-tumoral clonal T cell expansion (Figure 3D). Interestingly, ASPS and OS, which we also identified as having high relative enrichment of immune cell infiltrate and high median immune signature scores, were found to have the highest percentage of expanded intra-tumoral TCR clones, in 26.7% and 22.0% of patients, respectively. We did not find evidence of TCR clonal expansion in any tumor samples from *MYCN*-amplified NB, DSRCT, CCSK, ML, or WT in our cohort.

### Expression of cell-surface proteins, transcription factors, and tumor germline antigens

**Figure 4.**
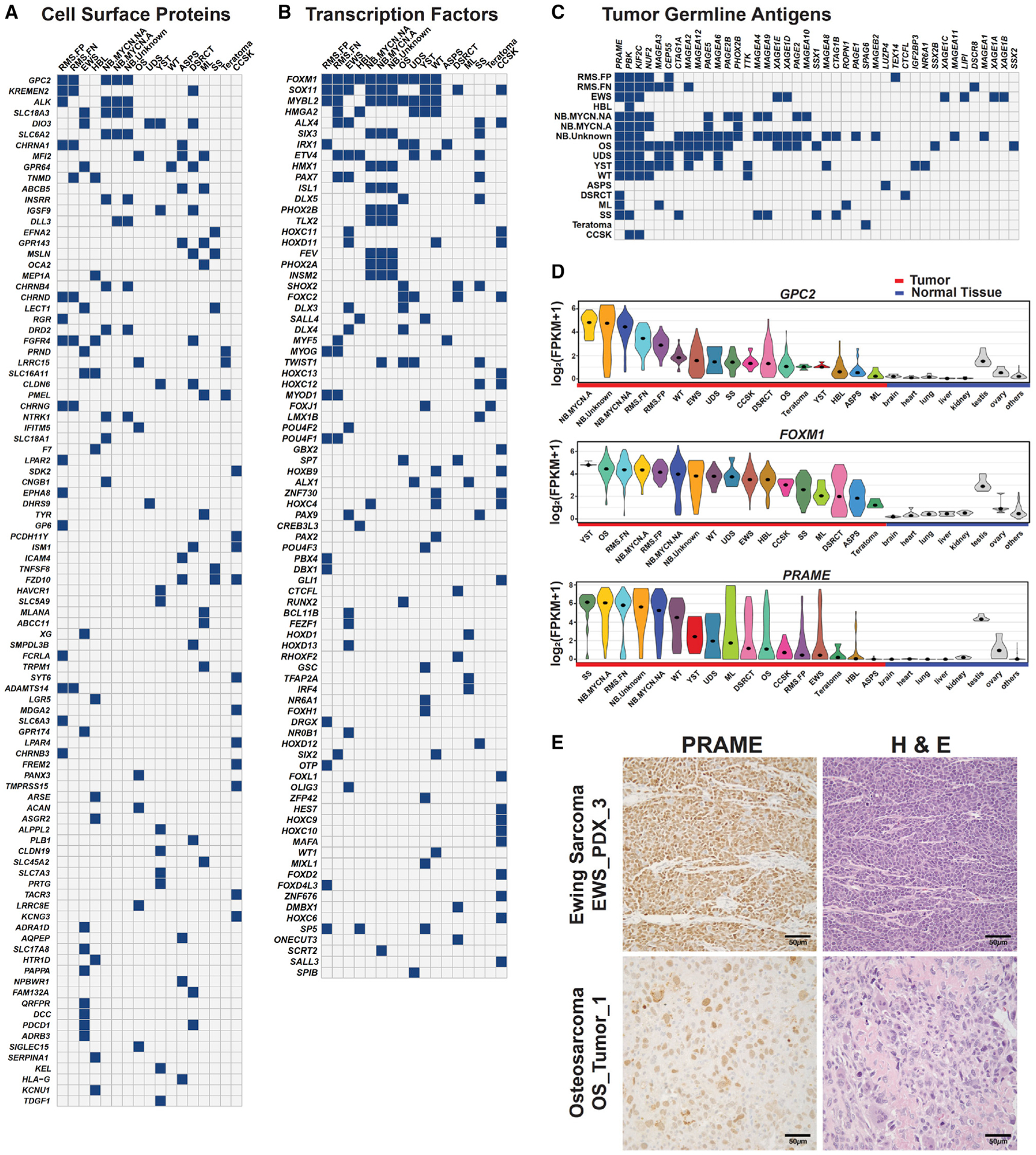
Tumor-specific gene expression (A–C) Tumor-specific gene expression including (A) cell-surface proteins, (B) transcription factors, and (C) tumor germline antigens. Fill indicates that the gene is overexpressed in the corresponding cancer type relative to normal tissues and has minimal expression in vital organs. (D) mRNA expression of top genes for each category in each cancer type, vital organs, testes, ovary, and other normal tissues. Dots represent the median expression for each cancer type. (E) Representative PRAME immunohistochemistry in Ewing sarcoma and osteosarcoma demonstrates a robust expression of PRAME protein in tumor cells. H&E, hematoxylin and eosin stain.

To discover potential targets for immunotherapy, we performed differential gene-expression analysis comparing each cancer type to 147 normal tissue samples (excluding testis and ovary) using stringent criteria of p ≤ 0.00001 with a fold change ≥16 compared to normal. We first identified cancer-specific cell-surface genes that may be targets for Chimeric Antigen Receptor T cell (CART) or antibody-based therapies. This approach identified 107 cell-surface genes (Figure 4A; Table S3A), with notable findings including *GPC2*, *ALK*, and *FGFR4*, which are being developed in preclinical or clinical studies as targets for CART therapies by our group and others (Richards et al., 2018). To identify other genes that may be a potential source of immunoreactivity for adoptive cellular therapy (ACT), we also performed a similar analysis for transcription factors and tumor germline antigens (TGAs) to identify those that are robustly expressed in tumors but show low or absent expression in normal tissues. We identified 88 transcription factors, many of which have been described previously as part of the core regulatory transcription factor circuitry in specific cancer types, such as *PHOX2B*, *TWIST1*, and *ISL1* in neuroblastoma (Durbin et al., 2018; Selmi et al., 2015); *MYOD1* and *MYOG* in rhabdomyosarcoma (Gryder et al., 2017); *NR0B1* in Ewing sarcoma (Kinsey et al., 2006) (Figure 4B; Table S3B). For TGAs, we identified 43 genes, including those that are currently under clinical investigation as ACT targets such as NY-ESO-1 (*CTAG1A*) in synovial sarcoma (D’Angelo et al., 2018b), and *PRAME* in a number of histologic types (Gutzmer et al., 2016; Luk et al., 2018) (Figure 4C; Table S3C). Although the median expression of these potential immunotherapeutic targets was high in various cancer types, their expression was not uniform across all tumors (Figure 4D).

To evaluate whether currently available cell line models exhibit expression of cancer-specific potential immunotherapy targets congruent to that observed in patient tumors, we evaluated transcriptome sequencing of a panel of 131 commonly utilized cell lines. For top hits *GPC2*, *FOXM1*, and *PRAME*, cell line expression was similar to the corresponding tumors of the same histology (Figure S6A). More broadly, we report the expression data for commonly used cell lines representative of their respective cancers for ASPS, EWS, NB.MYCN.A, NB.MYCN.NA, OS, RMS.FP, RMS.FN, and SS, enabling researchers to identify suitable cell lines for further study based on gene-expression level of specific immune targets (Table S3; https://omicsoncogenomics.ccr.cancer.gov/cgi-bin/JK).

Of tumor germline antigens, we identified PRAME as a potential multi-pediatric cancer target, expressed in many of the tumor types but showing minimal to no expression in all normal organs except testes and ovaries (Figures 4C and 4D). High protein expression of PRAME in neuroblastoma (Oberthuer et al., 2004) and osteosarcoma (Tan et al., 2012) have previously been reported. To validate expression in additional tumor types, we performed immunohistochemistry (IHC) of PRAME on an independent panel of pediatric solid tumors and patient derived xenografts (PDXs), which confirmed its robust expression in 7/12 (58%) samples (Figure S6D). Representative images are shown for OS, EWS, fusion-negative RMS, and control tissues (Figure 4E; Figure S6).

### Identification of targetable tumor antigens in OS and EWS using immunopeptidome

To further validate our RNA-seq findings of cancer-specific antigens and understand how these antigens might be presented by MHC class I complexes, we performed immunopeptidome (HLA-Ligandome) analysis using MHC class I immunoprecipitation (IP), peptide elution, and identification using LC-MS/MS on HLA*A2:01 positive cell lines (Figure S7). We focused on OS and EWS as these are among the most common pediatric sarcomas and remain a significant therapeutic challenge for metastatic disease. Integration of our transcriptomic and immunopeptidomic analyses revealed high-affinity peptides derived from tumor-associated genes such as PRAME, PBK, and several MAGE gene family genes (Table 2). Several of these MHC class I peptides were also observed in immune-responsive adult malignancies and have been successfully targeted using engineered T cells with adoptive cell transfer (Table 2; Table S4).

**Figure 5.**
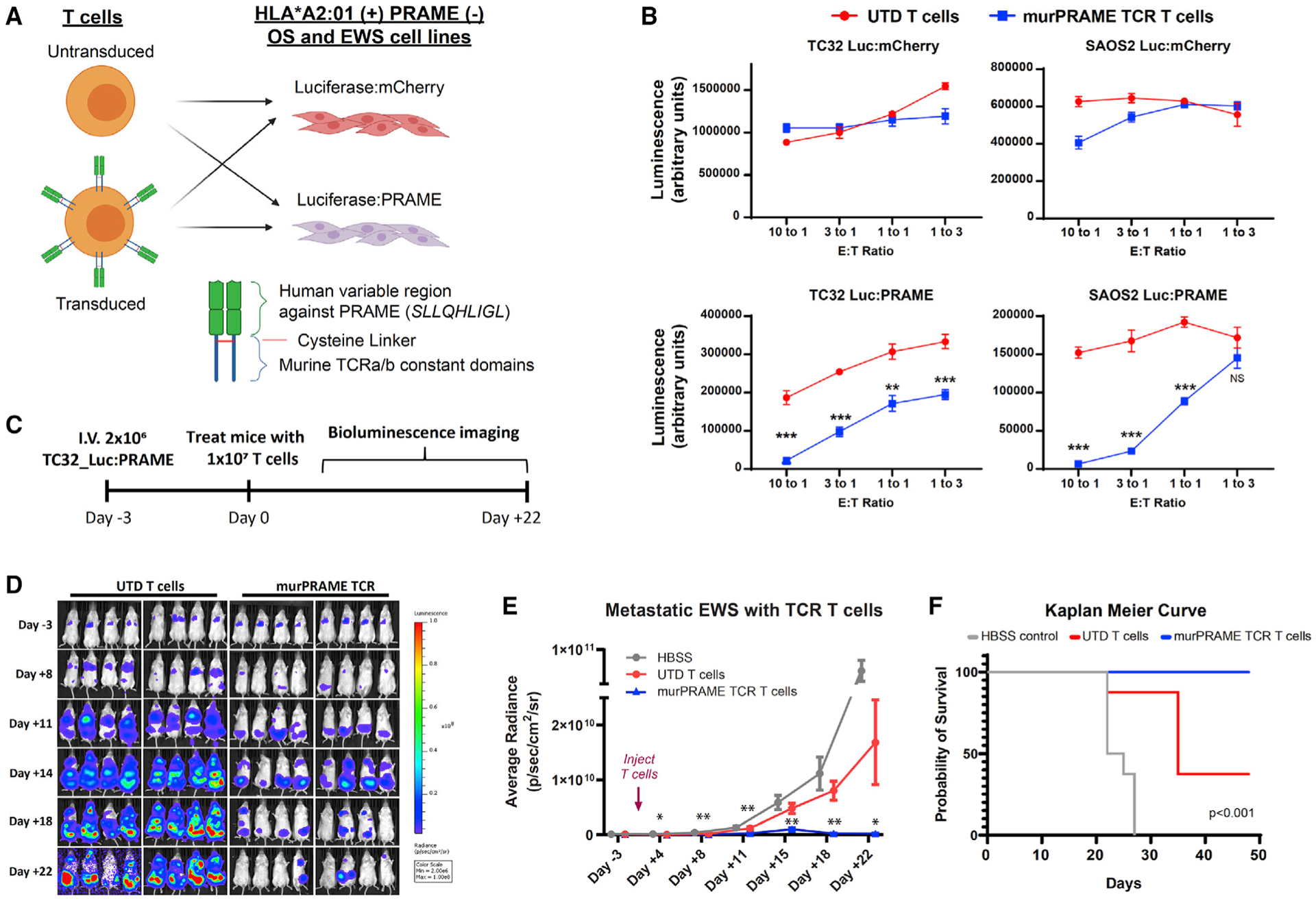
Specific anti-tumor activity of engineered T cells targeting a PRAME MHC class 1 peptide (A) Structure of engineered PRAME TCR and schema for testing the specificity and efficacy of murPRAME-TCR T cells. (B) *In vitro* co-culture of T cells with reporter cell lines at different effector:tumor (E:T) ratios. Luminescence was measured after 24 h of co-culture and reported as mean ± SEM (n = 3); **p < 0.01, ***p < 0.001. (C) Schema for treating metastatic EWS xenograft model with murPRAME-TCR T cells. (D) Bioluminescence images of TC32-Luc:PRAME cells after IV injection and treatment with vehicle or T cells. (E) Quantification of bioluminescence imagining reported as mean ± SEM (n = 8 per group). p values of UTD versus murPRAME-TCR mice displayed as *p < 0.05, **p < 0.01. (F) Kaplan-Meier analysis of mouse survival using log-rank test (n = 8 per group).

Among the peptides identified, we identified two HLA*A2:01 restricted peptides (*ALLPSLSHC*, *SLLQHLIGL*) derived from the PRAME protein (Table 2; Table S4). As a proof of concept for targeting PRAME, we tested a previously reported TCR targeting the PRAME HLA*A2:01 peptide *SLLQHLIGL* (Amir et al., 2011). Healthy donor T cells expressing the PRAME TCR showed cytotoxicity and cytokine production when co-cultured with U2OS (Figure S7), an osteosarcoma cell line that we found to present *SLLQHLIGL* on HLA*A2:01. To improve activity and specificity, we modified the PRAME TCR by swapping the TCR constant domains with the murine equivalent and adding cysteine linkers to improve exogenous TCR-α-TCR-β pairing (Cohen et al., 2006, 2007) (herein called murPRAME-TCR). We next engineered SAOS2 (OS) and TC32 (EWS) cells expressing a reporter luciferase:mCherry (negative control) or luciferase:PRAME (positive control) (Figure 5A). SAOS2 and TC32 cell lines were selected for their high expression of HLA*A2:01 (Figure S7A) and lack of endogenous PRAME expression, allowing us to test the specificity of the murPRAME-TCR. Co-culture of these lines with the murPRAME-TCR cells had no significant effect on either control cell line expressing luciferase:mCherry (Figure 5B, top). However, we observed significant cytotoxicity against SAOS2 and TC32 expressing PRAME from murPRAME-TCR T cells (Figure 5B, bottom).

Last, we tested whether murPRAME-TCR T cells could have therapeutic potential as an adoptive cell therapy *in vivo* using an aggressive metastatic EWS mouse model with intravenous delivery of PRAME-expressing TC32 cells (Figure 5C). After engraftment, mice were randomized and treated with vehicle (Hank’s balanced salt solution [HBSS]), untransduced T cells (UTD), or murPRAME-TCR-transduced T cells (Figure 5C). Saline and UTD-treated mice displayed rapid, disseminated tumor growth, whereas mice treated with murPRAME-TCR T cells had a significant and durable regression of their tumors and prolonged survival (Figures 5D–5F). Altogether, these data demonstrate that PRAME can be effectively targeted as an adoptive TCR cell therapy in these *in vitro* and *in vivo* models.

## DISCUSSION

Here, we report one of the largest and most comprehensive immune-transcriptomic landscape of extracranial pediatric solid tumors, with data derived from 788 malignant samples including 657 solid tumor samples across 14 diagnoses from 623 pediatric and young adult patients and 131 commonly used cancer cell lines. Success with checkpoint inhibitor therapy in many cancer types has led to an increased desire to understand the tumor-immune microenvironment interactions. Toward this end, we describe the immunogenomic landscape of our cohort of pediatric solid malignancies including tumor-infiltrating lymphocyte composition, cancer germline antigen and cell-surface protein expression, immune checkpoint expression, and T cell receptor repertoire. Notably, we observed a substantial degree of intra-histologic variance in all immunogenomic features evaluated, suggesting that histology alone may be insufficient for immunotherapeutic selection or trial design in these diseases. Broadly, we note a generally lower level of immune cell infiltration in most pediatric solid tumors compared to common adult tumors, with a notable exception of ASPS.

A high tumor mutation burden (TMB) has been associated with clinical response to checkpoint inhibition (Chan et al., 2019). Pediatric cancers typically have a low somatic mutation burden relative to common adult cancers (Gröbner et al., 2018; Ma et al., 2018); however, we have previously reported that the TMB in relapsed samples can increase two to three times compared to their primary tumors (Chang et al., 2016; Eleveld et al., 2015). Nonetheless, some cancers with low TMB such as clear cell renal cell carcinoma show a strong intratumor immune-related cytolytic activity and prominent immune infiltrate, findings that may be related to their clinical responsiveness to immune checkpoint inhibitors (Miao et al., 2018). These results emphasize that TMB may not be the only source of immunogenic triggers. Notably, ASPS, a disease recently found to be highly responsive to immune checkpoint inhibition (Wilky et al., 2019), was observed in our study to be among the highest degree of baseline TIL infiltrate despite being a low-TMB fusion-driven malignancy. Intra-tumoral T cell receptor clonality has been associated with clinical outcomes in metastatic cancers (Tumeh et al., 2014) and in the setting of immunotherapies (Zhang et al., 2020). Utilizing TCR prediction methodology to assess intra-tumoral clonotypes, we were able to demonstrate cases of robust expansion of TCR clones in 5.8% of patients, occurring at the highest frequency in ASPS and OS tumors, which suggests that a subset of pediatric solid tumors are more primed for immunotherapeutic interventions.

A striking finding from this study is the identification of high median T cell infiltration in OS relative to other pediatric solid tumors and a significant correlation between immune cell infiltrate and patient survival. We further show that CD8^+^ T cell infiltration is independent of metastasis, a known predictor of poor outcomes. Prior reports have also demonstrated robust immune cell infiltrate and PD-L1 expression in a subset of OS, though studies have conflicted results regarding the correlation between these immunologic features and patient outcomes (Thanindratarn et al., 2019; Wunder et al., 2020). In contrast to these immunologically favorable observations, we also observed pronounced expression of many additional immune inhibitory signaling molecules in osteosarcoma tumors, including *TGFB1* and *CSF1R*. Our findings are congruent with a previous analysis in osteosarcoma that utilizes a partially overlapping osteosarcoma cohort in the Therapeutically Applicable Research to Generate Effective Treatments (TARGET) dataset (Wu et al., 2020), which reported OS to be near the 50^th^ percentile compared to adult TCGA tumor types based on the rank order of tumors by median immune score, and multiple immune inhibitory pathways were also noted to be active. Given the limited efficacy of immune checkpoint inhibitor monotherapy for OS in clinical trials to date (D’Angelo et al., 2018a; Merchant et al., 2016; Tawbi et al., 2017), these results suggest potential co-inhibitory pathways in this disease that would make rationale targets for combination immunotherapies. (Song et al., 2021; Wu et al., 2020).

In addition to checkpoint blockade, directed immune targeting of tumor expressed antigens is another broad strategy for cancer immunotherapy. Toward this goal, we provide an overview of the expressed antigens in our cohort that are predicted to be the most translationally relevant due to differential expression from normal tissues. Our results confirm many of the tumor germline antigens that are in current clinical development, as well as a broad landscape of additional targets. As proof of concept of utilizing transcriptomics and immunopeptidomic approaches, we identified PRAME as an immune target, a highly differentially expressed protein with its peptide presented on the cell surface in the context of HLA-A2, the most frequent HLA allele in humans. Despite minor nonspecific cytotoxicity, which is commonly observed with infusion of UTD, our modified PRAME TCR-transduced T cells showed significantly higher *in vitro* activity in both OS and EWS cell line models, as well as significant potency *in vivo* in a metastatic EWS mouse model. Importantly, our data suggest that PRAME may be a broad, multi-cancer immunotherapy target in pediatric extracranial solid malignancies, similar to efforts in hematologic malignancies where clinical trials are underway (e.g., NCT02494167 and NCT02203903).

In summary, we describe here, one of the largest to date, transcriptomics-derived immunogenomics surveys of extracranial pediatric solid malignancies. We find significant correlations between immunogenomic features, such as immune cell infiltration, especially intra-tumoral clonal T cell infiltration, with patient survival. We provide a landscape of expressed tumor antigens that are most likely to be amenable to immunotherapeutic targeting. We further provide orthogonal confirmation of transcriptomic findings by evaluating the immunopeptidome of osteosarcoma and demonstrate proof of concept *in vitro* cytotoxicity using a transcriptomics-informed ACT approach. This work provides a critical framework for immune targeting of low mutational burden extracranial solid tumors using transcriptome profiling data. Furthermore, a companion gene-expression database (https://omics-oncogenomics.ccr.cancer.gov/cgi-bin/JK) derived from this study with outcome data including overall and event-free survival for osteosarcoma and neuroblastoma allows further exploration of this rich dataset. Finally, we report on an engineered TCR against PRAME can be developed for ACT immunotherapy for patients with pediatric solid tumors with HLA-A*02.

### Limitations of the study

A limitation of our study is the availability of clinical outcomes data in only two (NB and OS) tumor types studied. Despite this, we have reported a consistent association between high immune infiltrate and immune signatures score and favorable prognosis in both osteosarcoma and neuroblastoma (Wei et al., 2018), a result that remains significant even when accounting for metastatic disease status. Despite the consistency observed in these two tumor types, we cannot evaluate whether this is a universal finding among pediatric solid tumor types or specific to these two diseases.

## STAR★METHODS

### RESOURCE AVAILABILITY

#### Lead contact

Further information and requests for resources and reagents should be directed to and will be fulfilled by the lead contact, Javed Khan (khanjav@mail.nih.gov).

#### Material availability

All stable reagents generated in this study will be made available from the lead contact on request after a completed materials transfer agreement if there is potential for commercial application.

#### Data and code availability

- The raw RNA-seq data analyzed in this study are available from the public databases such as Gene Expression Omnibus (GEO; GSE89413 and GSE84629 for NBL cell lines and RD cell line respectively) or the database of Genotypes and Phenotypes (dbGaP; phs000466 for CCSK; phs000467 for NBL; phs000468 for OS; phs000720 for RMS, phs000768 for EWS; phs001052 for Omics study; and phs001928 for the remainder). The mass spectrometry proteomics data have been deposited to the ProteomeXchange Consortium via the PRIDE partner repository (https://www.ebi.ac.uk/pride) with the dataset identifier PXD017130. Accession numbers are listed in the key resources table. The expression data for this study are available at the Oncogenomics Expression Database (https://omics-oncogenomics.ccr.cancer.gov/cgi-bin/JK).
- Code of a custom bioinformatic pipeline to analyze RNA-seq data in this study is deposited at Zenodo (https://zenodo.org/record/5608456)). An R package for Kaplan Meier optimization is available from https://zenodo.org/record/5610858.
- Any additional information required to reanalyze the data reported in this study is available from the lead contact upon request.

### EXPERIMENTAL MODEL AND SUBJECT DETAILS

#### Human subjects

All human specimens for sequencing were obtained from patients with appropriate consent approved by the Institutional Review Board of the participating facilities and were deemed exempt by the Office of Human Subject Research. Tumors were classified by local pathological review using standard histologic techniques. For PRAME immunohistochemistry validation experiments, PDXs from diagnostic biopsies or surgical excisions were generated following written informed consent to participate in an institutional oncology specimen repository program; approval of these consents was obtained by the internal review board from Cincinnati Children’s Hospital Medical Center (CCHMC IRB approved protocol number 2008–0021). Samples in this study were all de-identified, therefore the age and gender of patients is not collected in this study. We performed RNA-seq on 935 samples in this study representing 657 pediatric extracranial solid tumors from 623 patients across 14 diagnoses and 147 normal human tissues.

#### Cell lines

We performed RNA-seq on 131 commonly used human pediatric cancer cell lines (Table S1). All cell lines used in this study have been authenticated by either STR profiling or genotyping by sequencing.

#### Mouse xenograft model

Female 6–8 weeks old NSG (NOD.Cg-Prkdc^scid^ Il2Rg^tm1Wjl/SzJ^) mice were used in Ewing sarcoma xenograft model to test antitumor activities for T cells expressing a modified TCR. Animal studies were approved by the NCI Animal Research Advisory Committee and conducted in accordance with Animal Welfare Regulations.

### METHOD DETAILS

#### RNA sequencing

Total RNAs was isolated from freshly frozen tumors using RNeasy mini kits or AllPrep DNA/RNA mini kits (QIAGEN, Germantown, MD). PolyA-selected or Ribozero-selected RNA libraries were prepared for RNA sequencing on Illumina HiSeq2000, 2500, and NextSeq500 according to the manufactures protocol (Illumina, San Diego, CA). Sequencer-generated bcl files were converted to fastq files using the bcl2fastq tool in *CASAVA* (Illumina, San Diego, CA) suite. Paired-end reads (100 bp for HiSeq2000, 125 bp for HiSeq2500, and 80 bp for NextSeq500) were assessed for quality using FastQC and the average mapped unique reads in this study is > 85 million for each sample (Table S1B). Fastq files were then mapped to GRCH37 reference genome using the *STAR/2.5.3a* alignment algorithm (Dobin et al., 2013) and subsequently quantified by RSEM program (Li and Dewey, 2011) based upon Ensembl GRCh37.75 gene annotation.

#### Single sample gene set enrichment analysis (ssGSEA)

Read counts for each gene between samples was normalized using TMM method implemented in edgeR (Robinson et al., 2010) and then transformed to FPKM. This expression data was used to predict enrichment scores of twenty-four immune signatures obtained from CIBERSORT (Newman et al., 2015) and ESTIMATE (Yoshihara et al., 2013) packages using single-sample GSEA (ssGSEA) from GenePattern (https://www.genepattern.org/modules/docs/ssGSEAProjection/4).

#### Kaplan Meier optimization

We used a KM optimization procedure, a computational technique for performing optimized Kaplan-Meier survival analysis on gene expression-derived signatures (Wei et al., 2018). Briefly, for a given gene signature, this procedure finds an optimal cutoff for stratifying patients into low-and high-risk groups that results in the maximal separation of the Kaplan-Meier survival curves and then estimates the statistical significance of this cutoff by means of the permutation test. This technique was implemented as an R package available from https://github.com/ibkstore/kmcut. We performed the KM optimization on the osteosarcoma patient cohort where outcome data were available.

#### Cancer-specific antigen analysis

Within each cancer diagnosis, gene expression of cell surface molecules, and cancer-specific transcription factors, tumor germline antigens, were compared against normal tissue samples with the matching library prep using edgeR (Robinson et al., 2010) package. The following criteria were used to select cancer-specific differentially expressed genes:

- Differential gene expression P value (compare to all normal except testis & Ovary) ≤ 0.00001
- Differential gene expression LogFC (compare to All normal except testis & Ovary) ≥ 4 (i.e., FC ≥ 16-fold)
- Expression in vital organs (Heart and Brain) < 1 FPKM
- Expression in Tumor ≥ 5 FPKM

#### T cell receptor analysis

Transcriptome sequencing reads from tumor samples were aligned to V, D, J and C genes of T cell receptors and subsequently assembled to extract TCR sequences using MiXCR 3.0.10 (Bolotin et al., 2015). Post processing of the later repertoire data was performed using VDJtools1.2.1 (Shugay et al., 2015). For downstream analysis, we considered only TRB-CDR3 amino acid sequences.

#### Multiplexed protein analysis

We validated our RNA-seq findings on an independent neuroblastoma tissue array (Saletta et al., 2017) using a multiplex protein detection assay (Nanostring). After deparaffinized and rehydrated, Formalin-fixed paraffin-embedded (FFPE) tissue cores were incubated with a cocktail of 61 primary antibodies which have their own unique and UV photocleavable indexing oligo, and 2 fluorescent markers (CD45 and Tyrosine). After overnight incubation, the slides were stained with Syto83 (nuclear DNA stain) for 15 mins before image processing using Nanostring GeoMx Digital Spatial Profiler. Slides were loaded onto the stage of an inverted microscope and wide field fluorescence imaging was performed with epi-illumination from visible LED light engine. 20× images from all the cores on the TMA were stitched together to yield a high-resolution image of the tissue area of interest. Regions of interest (ROIs) within 650μM diameter circle were selected based on the fluorescent image, and UV LED light was collimated to be reflected from the digital micromirror device (DMD) surface into the microscope objective and cleaved the oligoes from these primary antibodies. A microcapillary tip collected cleaved oligoes into the paired wells in a 96-well plate. 2 μL of these cleaved oligoes were further hybridized with Nanostring designed Tag-set. After overnight hybridization at 65°C in a thermocycler, samples were pooled by column and processed using the nCounter Prep Station and Digital Analyzer as per manufacturer instructions (Nanostring). Digital counts from tags corresponding to protein probes were analyzed using IgG isotype background subtraction and z-scored normalized.

#### Immunohistochemistry analysis

Formalin-fixed paraffin-embedded (FFPE) tissue blocks from primary bone osteosarcoma specimens were properly annotated by pathologist, cut into 5μm sections, and mounted onto plus-charge glass slides. For each specimen staining with CD3 (clone PS1; Leica Biosystems), CD8 (clone C8144B; Cell Marque), and CD163 (clone Novacastra10D6; Leica Biosystems) were performed according to standard protocols. IHC for PD-L1 (Spring Bioscience, clone SP142) at 0.096 mg/mL and isotype control (Spring Bioscience, clone SP137) at 1μg/mL were utilized(Sunshine et al., 2017). Whole slides scanning used Scanscope XT and digital pictures were taken via the Indica Labs HALO digital image analysis platform.

#### MHC class I flow cytometry

Five osteosarcoma cell lines were stained with antibodies which is pan-reactive for MHC class I (clone W6/32, BioLegend Cat. 311405) or specific for HLA*A2 (clone BB7.2, BioLegend, Cat. 343308). Flow cytometry was performed using BD LSRFortessa cell analyzer (BD Biosciences).

#### Peptide sequencing by tandem mass spectrometry

Osteosarcoma MHC class I bound peptides were isolated as described previously. Due to poor HLA*A2 IP efficiency using the serotype specific antibody (clone BB7.2), we synthesized a 3×FLAG-HLA*A2:01 lentiviral plasmid for HLA*A2:01 peptide identification. Briefly, 2–5×10^8^ cells were washed with ice-cold PBS and lysed in a mild lysis buffer (20mM Tris-HCL pH = 8.5, 100mM NaCl, 1 mM EDTA, 1% Triton X-100, 1:80 Halt Protease Inhibitor). Lysates were sonicated, clarified by centrifugation for 1 hour at 20,000 g at 4°C, and immunoprecipitated with anti-panMHC class I (clone W6/32, BioXcell, Cat. BE0079) or anti-FLAG (clone L5, BioXcell, Cat. 637304) coupled protein G agarose (ThermoFisher, Cat. 22851). MHC complexes were washed and eluted in 0.15% trifluoroacetic acid in water. MHC bound peptides were isolated using C_18_ solid phase extraction columns (Sigma, Cat. 52601-U) and further purified by C_18_ spin tips (ThermoFisher, Cat. 84850) according to the manufacturer’s instructions.

Purified HLA-associated peptides were sequenced by tandem mass spectrometry (MS) using an Orbitrap Q-Exactive HF mass spectrometer (Thermo Scientific, Waltham, MA). Briefly, peptides were separated on a reverse phase C_18_ Nano column (75μm × 250mm, 2 μm particle) using a 90-minute effective gradient with 4%–35% phase B (0.1% formic acid in acetonitrile) on an Ultimate 3000 Nano liquid chromatography (Thermo Scientific, Waltham, MA). Data dependent acquisition mode was used to profile the HLA peptidome. MS full scan range was 375–1650 m/z at resolution 120,000, and the top 15 most abundant peaks with assigned charge state 1–4 were selected for MS/MS fragmentation using high collision dissociation (HCD) at resolution 30,000 and maximum injection time was 200 ms, isolation window was 1.4 m/z, and dynamic exclusion was set to 20 s. The peptide sequence alignment was mapped by PEKAS studio (Tran et al., 2019) (Version 8.5, Bioinformatics Solutions Inc.) using UniProtKB human proteome sequence database (released on Feb 7^th^ 2017). In the PEAKS searching engine, no enzyme digestion (for natural peptides), the Orbi-Orbi HCD fragmentation, no variable modification, and *de novo* sequencing (library free search) were selected. The precursor mass tolerance was 15ppm and fragment ion tolerance was 0.5Da. The false discovery rate (FDR) was estimated by decoy-fusion database and was set to 5%. Selected peptides tandem mass spectrum was visualized and manually inspected to assure the data quality.

#### Modification of TCR against PRAME

We modified a TCR which recognizes the HLA*A2-restricted *SLLQHLIGL* peptide (Amir et al., 2011) by exchanging the constant domains of the *TRAV* and *TRBV* alleles with the murine equivalent TCR constant domains as previously described (Cohen et al., 2006). In addition, to increase the pairing between exogenous TCRα and TCRβ, cysteine substitutions were included in the murine constant domains of both the TRAV (T→C) and TRBV (S→C) positions 47 and 57 of the mouse constant domains, respectively (Cohen et al., 2007). After design, codon-optimized sequences were synthesized (Genscript) and cloned downstream of the EF1a promoter. Cleavable 2A peptide sequences were used for co-expression of TRAV, TRBV and truncated EGFR.

#### Generation of reporter cell lines

Stable expression of firefly luciferase and mCherry or PRAME was performed by lentiviral transduction of cells with pLenti_luciferase-p2a-mCherry or pLenti_luciferase-p2a-PRAMEv5tag lentivirus. In brief, lentiviral packaging was performed in LentiX-293T (Takara Clontech) cells by co-transfection with a luciferase transfer plasmid along with psPax2 (Addgene #12260) and pMD2.G (Addgene #12259). HLA*A2:01 positive, PRAME negative cell lines SAOS2 and TC32 were transduced with diluted lentiviral supernatant supplemented with polybrene. Stable expression was achieved by selection for geneticin for at least 7 days.

#### Generation of transduced T cells

Leukapheresis samples from two healthy donors were purchased from the NIH Blood Bank (Bethesda, MD). Peripheral blood mononuclear cells (PBMC) were isolated using Histopaque (Sigma, Cat. 10771). T cells were stimulated using CD3/CD8 Dynabeads (Invitrogen, Cat. 11131D) in the presence of IL-2 (40IU, PeproTech, Cat. 200–02). After 48 hours of stimulation, T cells were transduced with a lentiviral vector containing truncated EGFR and murPRAME-TCR. Transduction efficiency was determined 4 days after transduction by flow cytometry using a tEGFR antibody (BioLegend, Cat. 352903).

#### *In vitro* T cell cytotoxicity assays and cytokine quantification

T cells (day 9 post stimulation) were co-cultured with 5,000 target cells in 96-well plates. T cell to target cell ratio (E:T) was determined based on transduction efficiency. After 24 hours, luciferase activity was measured using Steady-Glo Luciferase Assay (Promega, Cat. E2520). Experiment was performed in triplicate. For U2OS cytotoxicity assays, U2OS cells were seeded in 96 well plates and allowed to attach for 6 hours. Relative cell confluency was quantified using the xCELLigence RTCA MP instrument (ACEA biosciences, Inc.). Media or T cells were then added to wells at an effector:tumor ratio of 3:1. Data were acquired at 15-minute increments for 30 hours and an unpaired t test at the final time point was used for statistical analysis. For cytokine analyses, effector and target cells were co-cultured at a 3:1 ratio for 18 hours. Supernatants were collected and IFNγ was quantified using the V-PLEX human cytokine assay (Meso Scale Diagnostics, Cat. K151AOH-2).

#### Ewing Sarcoma xenograft model with adoptive T cell transfer

Animal studies were approved by the NCI Animal Research Advisory Committee and conducted in accordance with Animal Welfare Regulations. For xenograft studies, 2 × 10^6^ human Ewing sarcoma cell line TC32 expressing luciferase and PRAME were injected via tail vein into NSG mice. Tumor burden was monitored by bioluminescence imaging using the Perkin Xenogen IVIS Imaging System with 150mg/kg D-Luciferin (PerkinElmer, Cat. 122799–5). Mice were imaged 3 hours after TC32 injection to acquire baseline luminescence and then randomized into 3 groups treated with saline (n = 8), untransduced T cells (n = 8), or murPRAME-TCR T cells (n = 8). A total of 10^6^ UTD or murPRAME-TCR T cells were injected via tail vein for the T cell treatment groups. Unpaired t tests were performed at each time point comparing bioluminescence between UTD and murPRAME-TCR T cell treated mice. Kaplan Meier analysis was performed using GraphPad PRISM using a three-way log-rank test.

### QUANTIFICATION AND STATISTICAL ANALYSIS

Quantification and statistical analyses used in this study is described in detail in the previous section. Differential gene expression was quantified using edgeR (Robinson et al., 2010) package. Log-rank tests were performed in survival analyses using GraphPad PRISM. One-factor and two factor Cox regression analysis of overall survival was used to examine if immunological features derived from RNA-seq data are independent of tumor metastasis, a known outcome predictor. Cell viability or growth was measured using Steady-Glo Luciferase Assay (Promega, Cat. E2520) or relative cell confluency using the xCELLigence RTCA MP instrument (ACEA biosciences, Inc.) respectively. Tumor burden in mice was quantified by bioluminescence imaging using the Xenogen IVIS Imaging System (PerkinElmer). Unpaired Student’s t test was used in T cell cytotoxicity, cytokine release, and monitoring tumor burden experiments.

### ADDITIONAL RESOURCES

Accompanying this analysis, we provide a companion **Oncogenomics expression** database (https://omics-oncogenomics.ccr.cancer.gov/cgi-bin/JK) for all tumors, normal tissues, and commonly utilized cell lines in the research community. The data include gene expression derived from the RNA-seq together with outcome data for osteosarcoma and neuroblastoma with capabilities for Kaplan–Meier survival analysis and further data exploration.

Three databases are displayed:

1. Landscape - NCI; contains all RNA-seq data across all tumors, cell lines, and normal organs.
2. Landscape Neuroblastoma TARGET; Contains RNA-seq data for all Neuroblastoma tumors and cell lines.
3. Landscape Osteosarcoma TARGET; Contains RNA-seq data for all Osteosarcoma tumors and cell lines.

It allows users to search for individual genes of interest. The query results include heat-map and bar chart representations as well as link-outs to more detailed annotations. As all datasets can be visualized as raw log2 (FPKM-TMM normalized) or pre-normalized using Z-score or median centering. All query results can be downloaded as text files. Gene set enrichment analyses (GSEA) against lists of both curated and custom gene sets can be done at 1) Individual Sample where genes are ranged by the expression of all genes in that sample, or 2) genes ranked by correlation with that gene. Landscape Neuroblastoma TARGET and Landscape Osteosarcoma TARGET databases contain event-free and overall survival where the user can perform Kaplan-Meier analysis using median expression level for that gene or through a KM optimization process to identify the optimal threshold for gene expression as described above.

## Supplementary Material

1

2

3

4

5

## Figures and Tables

**Table 1.** Sample cohort

	Tumor samples			Normal samples	
Diagnosis and subtype	Tumor count	Cell line count	Subtotal	Tissue type	Count
Alveolar soft part sarcoma (ASPS)	16	3	19	adrenal gland	8
Clear cell sarcoma of kidney (CCSK)^a^	16	0	16	bladder	8
Desmoplastic small round cell tumor (DSRCT)	40	0	40	cerebellum	10
Ewing sarcoma (EWS)^b^	79	43	122	cerebrum	8
Hepatoblastoma (HBL)	30	0	30	colon	9
Melanoma (ML)	6	0	6	heart	7
Neuroblastoma (NB)^c^				ileum	8
MYCN-amplified (NB.MYCN.A)	47	28	75	kidney	8
MYCN-not amplified (NB.MYCN.NA)	147	12	159	liver	8
Unknown (NB.Unknown)	12	0	12	lung	8
Osteosarcoma (OS)^d^	93	9	102	muscle	5
Rhabdomyosarcoma (RMS)^e^				ovary	5
Fusion-negative (RMS-FN)	60	21	81	pancreas	6
Fusion-positive (RMS-FP)	38	12	50	prostate	9
Synovial sarcoma (SS)	31	3	34	skeletal muscle	6
Teratoma	4	0	4	spleen	5
Undifferentiated sarcoma (UDS)	6	0	6	stomach	7
Wilms tumor (WT)	27	0	27	testis	7
Yolk sac tumor (YST)	5	0	5	thyroid	1
				ureter	7
				uterus	7
Total	657	131	788		147

a Data are available in dbGaP: phs000466.

b Data are available in dbGaP: phs000768.

c Data are available in dbGaP: phs000467.

d Data are available in dbGaP: phs000468.

e Data are available in dbGaP: phs000720.

**Table 2.** Summary table of OS and EWS MHC class I peptides

Gene	Peptide	HLA allele specificity	Percentile rank	Predicted affinity (IC_50_ nM)	Other cancers presenting same peptide
PRAME	ALLPSLSHC	HLA-A*02:01	2.72	467.3	melanoma (Gloger et al., 2016)
	SLLQHLIGL	HLA-A*02:01	0.12	9.9	melanoma (Gloger et al., 2016; Bassani-Sternberg et al., 2016)
MAGEA1	KVLEYVIKV	HLA-A*02:01	0.05	5.9	Melanoma (Bassani-Sternberg et al., 2016; Pritchard et al., 2015)
MAGEA1/A4/A8	AETSYVKVL	HLA-B*44:02	0.09	72.4	melanoma (Pritchard et al., 2015), breast cancer (Ternette et al., 2018)
MAGEB2	GVYDGEEHSV	HLA-A*02:01	2.44	386.5	chronic myeloid leukemia (Hassan et al., 2013)
MAGED2	NADPQAVTM	HLA-C*05:01	0.08	172.1	breast cancer (Ternette et al., 2018; Rozanov et al., 2018), chronic lymphocytic leukemia (Nelde et al., 2018)
PBK	SYQKVIELF	HLA-C*07:04	0.65	7398.1	breast cancer (Ternette et al., 2018; Rozanov et al., 2018), glioblastoma (Shraibman et al., 2019), melanoma (Pritchard et al., 2015; Bassani-Sternberg et al., 2017)
KREMEN2	ALGPPGAAL	HLA-A*02:01	2.80	494.5	none reported
ULBP3	LLFDWSGTGRA	HLA-A*02:01	2.74	473.5	colon cancer (Bassani-Sternberg et al., 2015)
	LLFDWSGTGRADA	HLA-A*02:01	2.81	498.4	colon cancer (Bassani-Sternberg et al., 2015)
IGF2BP3	KIQEILTQV	HLA-A*02:01	0.23	17.7	melanoma (Gloger et al., 2016; Jensen et al., 2018), chronic myeloid leukemia (Jensen et al., 2018), colon cancer (Murphy et al., 2019), breast cancer (Ternette et al., 2018)
KIF20b	AEIEDIRVL	HLA-B*44:02	0.12	94.8	melanoma (Koumantou et al., 2019), chronic lymphocytic leukemia (Nelde et al., 2018)

A summary table of high-affinity peptides in OS and EWS cells. Peptide percentage ranks and predicted HLA affinities were calculated using NetMHC (Jurtz et al., 2017) with sequencing-identified HLA allele variants present in the corresponding cell line.

**Table:** KEY RESOURCES TABLE

Reagent or resource	Source	Identifier
Antibodies		
Anti-CD3	Leica Biosystems	Clone PS1
Anti-CD8	Cell Marque	Clone C8144B
Anti-CD163	Leica Biosystems	Clone Novasasta10D6
Anti-PD-L1	Spring Bioscience	Clone SP142
Anti-PD-L1 isotype control	Spring Bioscience	Clone SP137
Pan-reactive for MHC class I, clone W6/32	BioLegend	Cat. 311405
Anti-HLA*A2, clone BB7.2	BioLegend	Cat. 343308
Anti-FLAG, clone L5	BioXcell	Cat. 637304
Anti-tEGFR	BioLegend	Cat. 352903
Anti-PRAME	Abcam	Cat. ab219650
Chemicals, peptides, and recombinant proteins		
TruSeq Stranded mRNA library Prep kits	Illumina	https://www.illumina.com/products/by-type/sequencing-kits/library-prep-kits/truseq-stranded-mrna.html
TruSeq Stranded Total RNA	Illumina	https://www.illumina.com/products/by-type/sequencing-kits/library-prep-kits/truseq-stranded-total-rna.html
XenoLight D-Luciferin Potassium Salt	Perkin Elmer	Cat. 122799–5
Critical commercial assays		
Nanostring GeoMx Digital Spatial Profiler (DSP)	Nanostring	https://www.nanostring.com/products/geomx-digital-spatial-profiler/geomx-dsp-overview/
V-PLEX human cytokine assay	Meso Scale Diagnostics	Cat. K151AOH-2
Steady-Glo® Luciferase Assay System	Promega	E2520
Deposited data		
dbGAP phs001928 for pediatric cancers	This study	https://www.ncbi.nlm.nih.gov/gap/
dbGaP phs000466 for CCSK	(Gooskens et al., 2015)	https://www.ncbi.nlm.nih.gov/gap/
dbGaP phs000467 for NBL	(Wei et al., 2018)	https://www.ncbi.nlm.nih.gov/gap/
dbGaP phs000468 for OS	https://ocg.cancer.gov/programs/target/projects/osteosarcoma	https://www.ncbi.nlm.nih.gov/gap/
dbGaP phs000720 for RMS	(Shern et al., 2014)	https://www.ncbi.nlm.nih.gov/gap/
dbGaP phs000768 for EWS	(Brohl et al., 2014)	https://www.ncbi.nlm.nih.gov/gap/
dbGaP phs001052 for Omics study	(Chang et al., 2016)	https://www.ncbi.nlm.nih.gov/gap/
Gene expression of neuroblastoma cell lines; GEO GSE89413	(Harenza et al., 2017)	https://www.ncbi.nlm.nih.gov/geo/query/acc.cgi?acc=GSE89413
Immunopeptidomes data osteosarcoma cell lines	https://www.ebi.ac.uk/pride	Accession: PXD017130
Experimental models: Cell lines		
ASPS4c159	This study	N/A
CC-A	This study	N/A
FUUR1	This study	N/A
NCIEWS5000	This study	N/A
6647	This study	N/A
A673	This study	N/A
CHLA258	This study	N/A
CHLA352	This study	N/A
CHP100L	This study	N/A
SKES1	This study	N/A
SKNLO	This study	N/A
SKNMC	This study	N/A
TC106	This study	N/A
TC138	This study	N/A
TC167	This study	N/A
TC177	This study	N/A
TC215	This study	N/A
TC233	This study	N/A
TC244	This study	N/A
TC248	This study	N/A
TC253	This study	N/A
TC32	This study	N/A
TC487	This study	N/A
TC4C	This study	N/A
TTC466	This study	N/A
TTC475	This study	N/A
TTC547	This study	N/A
CHP134	This study	N/A
GILIN	This study	N/A
IMR32	This study	N/A
IMR5	This study	N/A
KCNR	This study	N/A
LAN1	This study	N/A
LAN5	This study	N/A
NB1691	This study	N/A
SKNBE2	This study	N/A
SKNDZ	This study	N/A
NBEB	This study	N/A
SHSY5Y	This study	N/A
SKNAS	This study	N/A
SKNFI	This study	N/A
SKNSH	This study	N/A
BIRCH	This study	N/A
CT-10	This study	N/A
CTR	This study	N/A
RD	This study	N/A
RH1	This study	N/A
RH18	This study	N/A
RMS559	This study	N/A
TTC-442	This study	N/A
TTC-516	This study	N/A
CW9109	This study	N/A
JR	This study	N/A
MP4	This study	N/A
NCI-ARMS1	This study	N/A
NCI-RMS-052	This study	N/A
RH28	This study	N/A
RH30	This study	N/A
RH4	This study	N/A
RH41	This study	N/A
RH5	This study	N/A
ASPS1	This study	N/A
CHLA10	This study	N/A
CHLA25	This study	N/A
CHLA32	This study	N/A
CHLA9	This study	N/A
ES8	This study	N/A
RDES	This study	N/A
TC71	This study	N/A
HOS	This study	N/A
SAOS2	This study	N/A
SJSA1	This study	N/A
U2OS	This study	N/A
Hs729	This study	N/A
RH36	This study	N/A
SKNEP1	This study	N/A
SKPNETLI	This study	N/A
TC240	This study	N/A
7556	This study	N/A
JR1	This study	N/A
RH2	This study	N/A
RH3	This study	N/A
RMS-YM	This study	N/A
RUCH2	This study	N/A
RUCH3	This study	N/A
SCMC	This study	N/A
T91–95	This study	N/A
TE617	This study	N/A
H170	This study	N/A
HR	(Linardic et al., 2007)	N/A
COGE352	(Teicher et al., 2015)	N/A
ES1	(Teicher et al., 2015)	N/A
ES2	(Teicher et al., 2015)	N/A
ES3	(Teicher et al., 2015)	N/A
ES4	(Teicher et al., 2015)	N/A
ES6	(Teicher et al., 2015)	N/A
ES7	(Teicher et al., 2015)	N/A
EW8	(Teicher et al., 2015)	N/A
CHA59	(Teicher et al., 2015)	N/A
KHOS240S	(Teicher et al., 2015)	N/A
KHOS312H	(Teicher et al., 2015)	N/A
KHOSNP	(Teicher et al., 2015)	N/A
OHS	(Teicher et al., 2015)	N/A
HSSY11	(Teicher et al., 2015)	N/A
SW982	(Teicher et al., 2015)	N/A
SYO1	(Teicher et al., 2015)	N/A
CHP-212	(Harenza et al., 2017)	N/A
COGN415	(Harenza et al., 2017)	N/A
COGN440	(Harenza et al., 2017)	N/A
COGN453	(Harenza et al., 2017)	N/A
COGN471	(Harenza et al., 2017)	N/A
COGN496	(Harenza et al., 2017)	N/A
COGN519	(Harenza et al., 2017)	N/A
COGN561	(Harenza et al., 2017)	N/A
COGN573	(Harenza et al., 2017)	N/A
KELLY	(Harenza et al., 2017)	N/A
NB1	(Harenza et al., 2017)	N/A
NB1643	(Harenza et al., 2017)	N/A
NBSD	(Harenza et al., 2017)	N/A
NGP	(Harenza et al., 2017)	N/A
NLF	(Harenza et al., 2017)	N/A
NMB	(Harenza et al., 2017)	N/A
SMSKAN	(Harenza et al., 2017)	N/A
SMSSAN	(Harenza et al., 2017)	N/A
COGN534	(Harenza et al., 2017)	N/A
COGN549	(Harenza et al., 2017)	N/A
FELIX	(Harenza et al., 2017)	N/A
LAN6	(Harenza et al., 2017)	N/A
NB16	(Harenza et al., 2017)	N/A
NB69	(Harenza et al., 2017)	N/A
NBLS	(Harenza et al., 2017)	N/A
Experimental models: Organisms/strains		
NOD.Cg-Prkdc^scid^ Il2Rg^tm1Wjl/SzJ^ (“NSG”) mice	https://www.jax.org/strain/005557	Stock No: 005557
Recombinant DNA		
Lentiviral expression construct of a TCR which recognizes the HLA*A2-restricted *SLLQHLIGL* peptide corresponding to PRAME	(Amir et al., 2011)	N/A
Lentiviral expression construct of luciferase and mCherry	This study	N/A
Lentiviral expression construct of luciferase and PRAME cDNA with C-term V5 tag	This study	N/A
Software and algorithms		
NGS bioinformatic pipeline	This study	https://zenodo.org/record/5608456
R (3.3.1)		https://www.r-project.org/
CASAVA	Illumina	RRID:SCR_001802; https://support.illumina.com/sequencing/sequencing_software/bcl2fastqconversion-software.html
STAR (2.5.3a)	(Dobin et al., 2013)	https://github.com/alexdobin/STAR
GATK (3.8–1)	(McKenna et al., 2010)	https://gatk.broadinstitute.org
Tophat-Fusion (2.0.13)	(Kim and Salzberg, 2011)	https://ccb.jhu.edu/software/tophat/fusion_index.shtml
FusionCatcher (1)	(Nicorici et al., 2014)	https://github.com/ndaniel/fusioncatcher
Star Fusion (1.3.1)	(Haas et al., 2017)	N/A
ssGSEA	(Barbie et al., 2009)	https://www.genepattern.org/modules/docs/ssGSEAProjection/4
IntegrateNeo (1.2.0)	(Zhang et al., 2017)	https://github.com/ChrisMaherLab/INTEGRATE-Neo
EdgeR (3.7)	(Robinson et al., 2010)	https://bioconductor.org/packages/release/bioc/html/edgeR.html
MiXCR (3.0.10)	(Bolotin et al., 2015)	https://mixcr.readthedocs.io/en/latest/
vdjTools (1.2.1)	(Shugay et al., 2015)	https://vdjtools-doc.readthedocs.io/en/master/
pVACTools (1.5.4)	(Hundal et al., 2020)	https://pvactools.readthedocs.io/en/latest/index.html
Superheatmap (0.1.0)	(Barter and Yu, 2018)	https://cran.r-project.org/web/packages/superheat/index.html
KM optimization	(Wei et al., 2018)	https://zenodo.org/record/5610858
PEAKS Studio	Bioinformatics Solutions Inc.	N/A
Other		
cBioPortal	Memorial Sloan Kettering Cancer Center, New York, NY	http://www.cbioportal.org/
VDJdb web browser	(Shugay et al., 2015)	https://vdjdb.cdr3.net/
TCRb2010	(Warren et al., 2011)	ftp://ftp.bcgsc.ca/supplementary/TCRb2010/
Oncogenomics Expression Database	This study	https://omics-oncogenomics.ccr.cancer.gov/cgi-bin/JK

## Highlights

- We describe the immunogenomics landscape of 14 pediatric extracranial solid tumors
- Immunophenotype is prognostic of survival in neuroblastoma and osteosarcoma
- We identify multiple targets for immune therapy
- Immune targeting of *PRAME* demonstrates *in vitro* and *in vivo* cytotoxicity
