# Supplementary material for: Immuno-transcriptomic profiling of extracranial pediatric solid malignancies
Source: Cell Rep. Author manuscript; Available in PMC 2021 Dec 4. (PMC8642810; doi:10.1016/j.celrep.2021.110047)
Supplement: 1 [file NIHMS1759318-supplement-1.pdf]

Cell Reports, Volume *37*

Supplemental information

Immuno-transcriptomic profiling

# of extracranial pediatric solid malignancies

Andrew S. Brohl, Sivasish Sindiri, Jun S. Wei, David Milewski, Hsien-Chao Chou, Young K. Song, Xinyu Wen, Jeetendra Kumar, Hue V. Reardon, Uma S. Mudunuri, Jack R. Collins, Sushma Nagaraj, Vineela Gangalapudi, Manoj Tyagi, Yuelin J. Zhu, Katherine E. Masih, Marielle E. Yohe, Jack F. Shern, Yue Qi, Udayan Guha, Daniel Catchpoole, Rimas J. Orentas, Igor B. Kuznetsov, Nicolas J. Llosa, John A. Ligon, Brian K. Turpin, Daniel G. Leino, Shintaro Iwata, Irene L. Andrulis, Jay S. Wunder, Silvia R.C. Toledo, Paul S. Meltzer, Ching Lau, Beverly A. Teicher, Heather Magnan, Marc Ladanyi, and Javed Khan

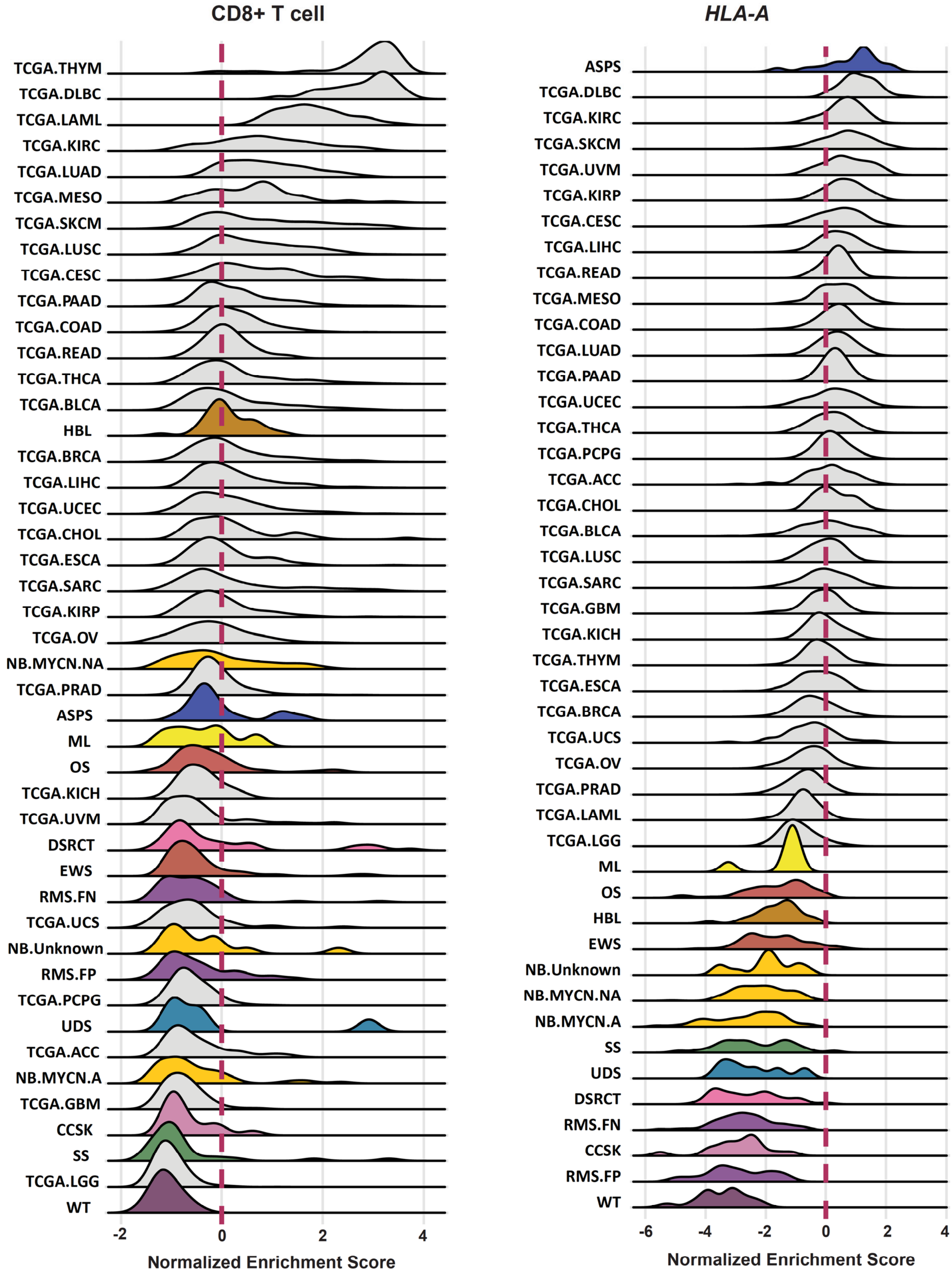

Figure S1. Distribution of CD8+ T cell enrichment, and HLA-A expression across cancer types including adult TCGA cancers (grey) co-analyzed with the study tumor cohort. Related to Figure 1.

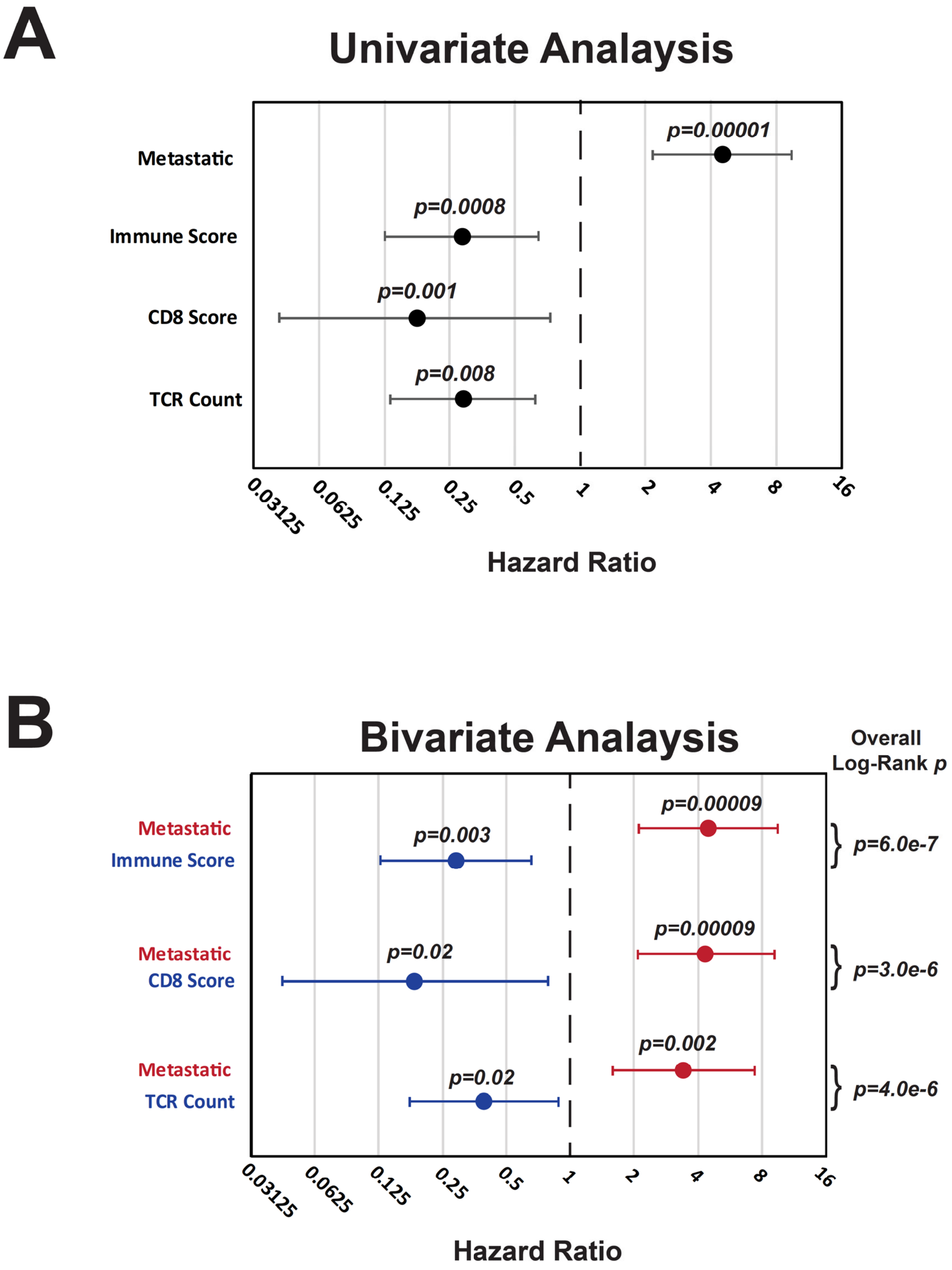

Figure S2. Immunological characteristics are independent predictors of metastatic status in osteosarcoma patients. One-factor (A) and two factor (B) Cox regression analysis of overall survival against immunologic characteristics and metastatic status demonstrates that high immune score, high CD8 enrichment score, and high TCR count are significantly associated with favorable prognosis independent from the metastatic status. Related to Figures 1 and 3.

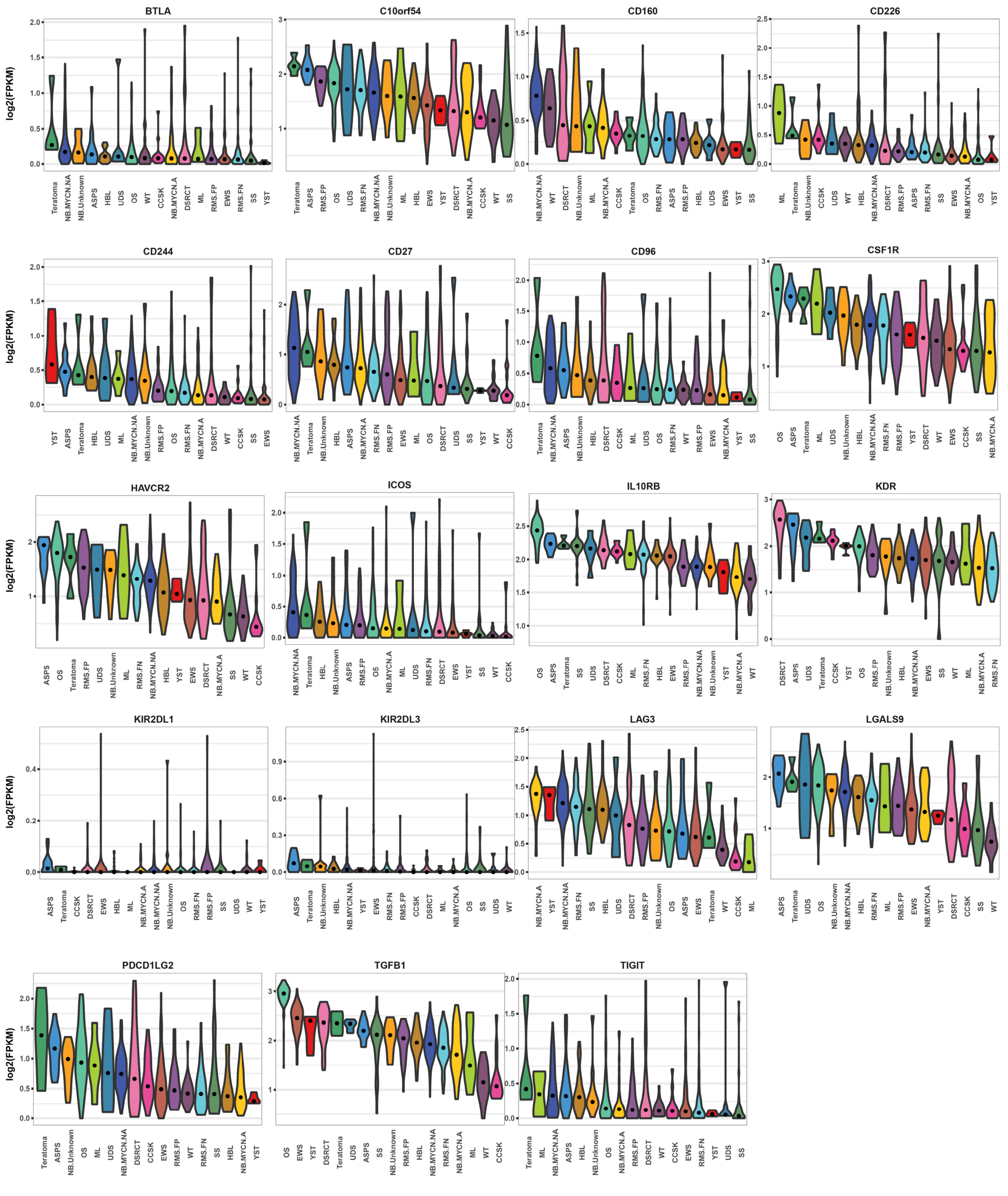

Figure S3. Expression of immune checkpoint and immune modulatory genes in pediatric solid tumors. Dots represent the median expression for each cancer type. Related to Figures 2 and S4.

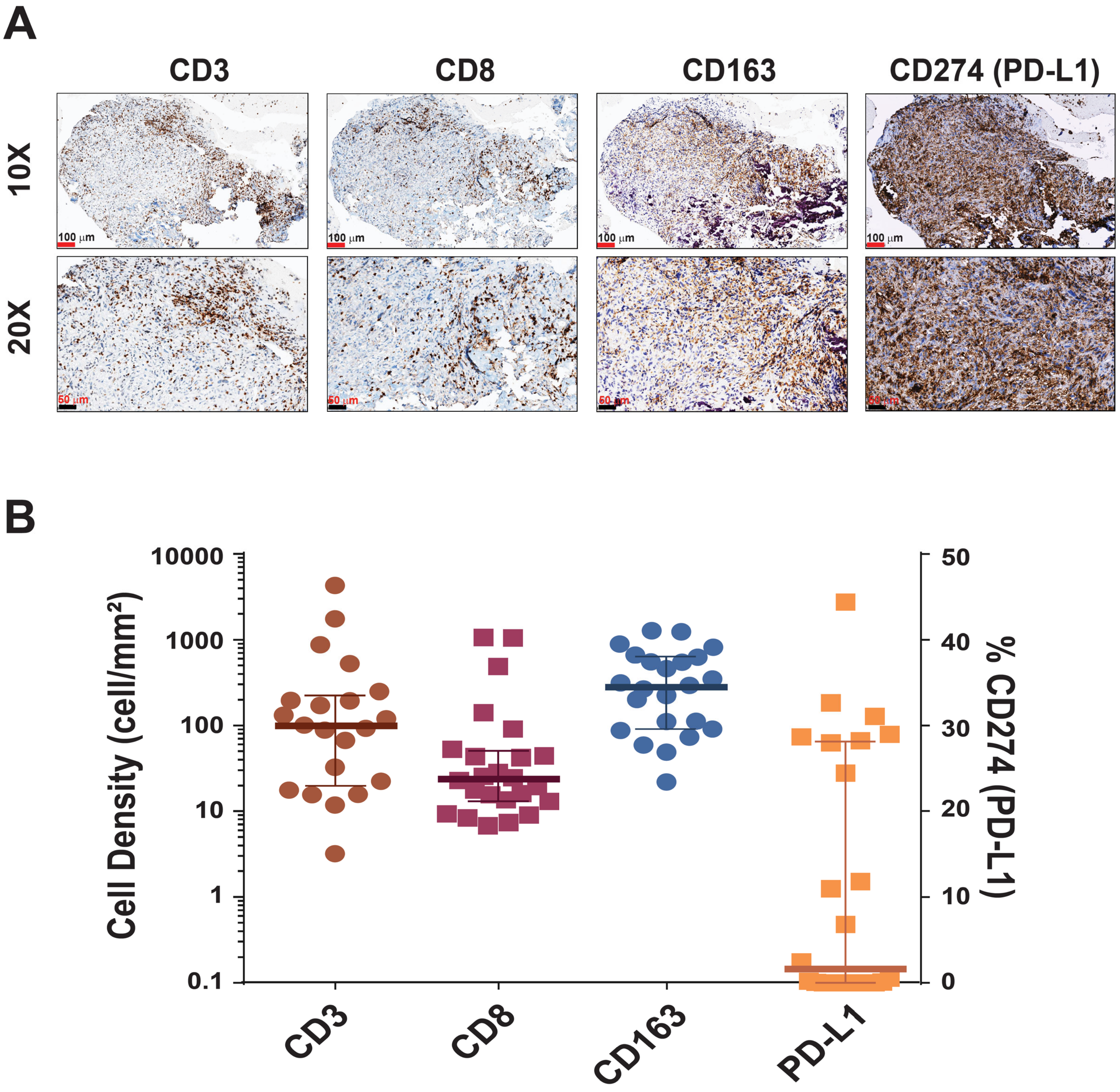

Figure S4. Immunohistochemistry (IHC) validation of the presence of immune cells and immune checkpoint expression as predicted by RNAseq in OS tumors. (A) Representative immunohistochemistry images from an osteosarcoma specimen demonstrate robust CD3+ T-cell, CD8+ cytotoxic T-cell, and CD163+ monocyte/macrophage infiltrates as well as CD274 (PD-L1) expression, consistent with our RNA-seq data. Red scale bars represent 100µm and black bars 50µm. **(B)** Quantification of immune cells using IHC in an independent osteosarcoma cohort (n=25) displayed heterogeneous levels of tumor-infiltrating immune cells. Thick lines represent medians and thin lines interquartile. Prominent PD-L1 expression (>1%) was seen in 12/24 (50%) evaluable tumors. Related to Figures 2 and S3.

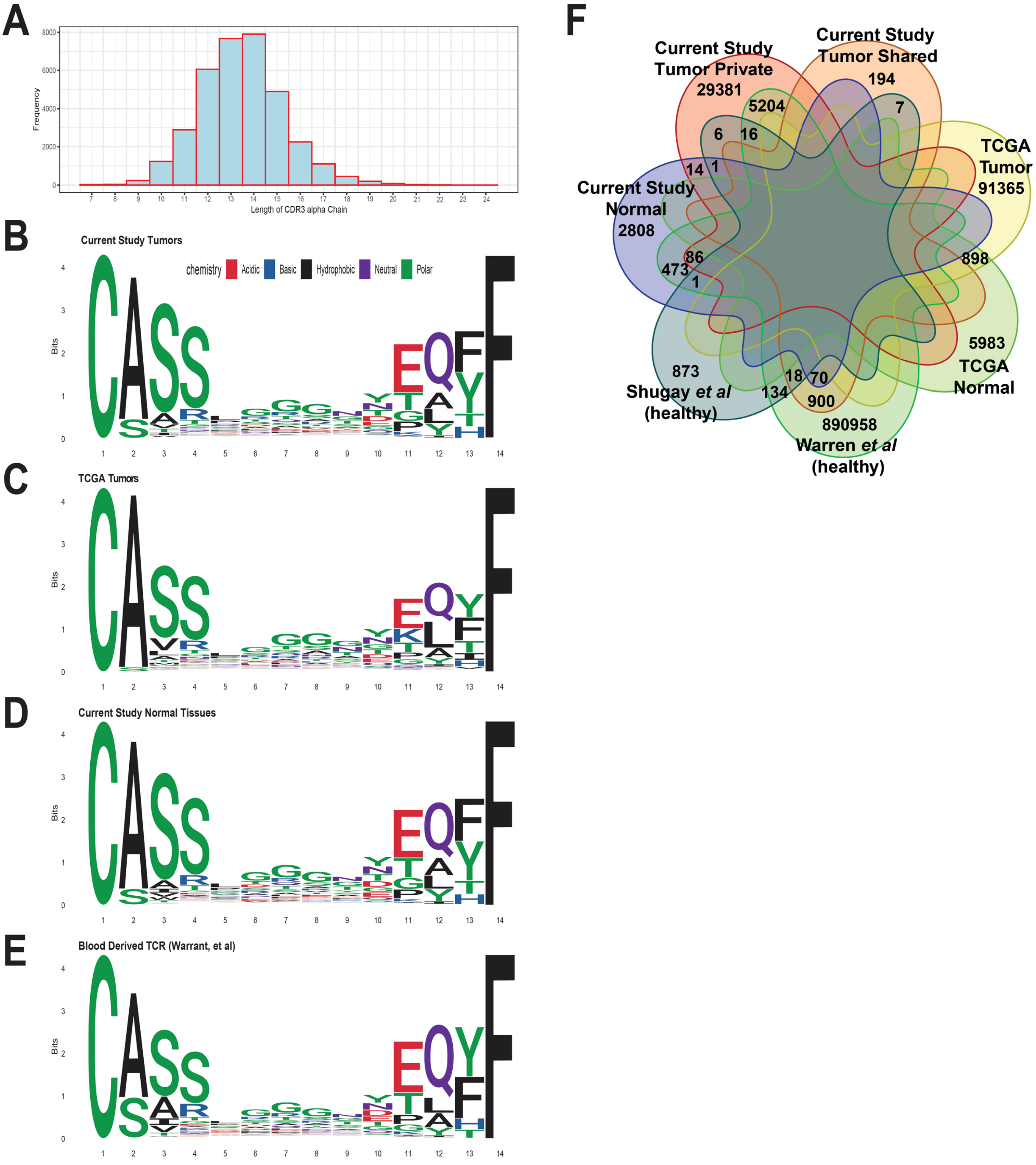

Figure S5. Characterization of T cell receptors (TCRs) using unique complementary-determining region 3 (CDR3) sequences. **(A)** CDR3 length distribution across the current study tumor cohort. **(B-E)** Amino acid usage in predicted CDR3 regions displayed with Weblogo software for 14 amino acid length CDR3 for the current study tumors **(B)**, across TCGA tumors **(C)**, and normal tissues in the current study **(D)**, and blood derived TCRs **(E)** (Warren et al., 2011). **(F)** Vein diagram depicting overlap of TCR nucleic acid sequences identified in our tumor cohort to those identified in TCGA as well as two healthy population databases. "Tumor Private" refers to TCRs identified in a single tumor within our cohort, and "Tumor Shared" refers to TCRs identified in >1 tumor within our cohort. Related to Figure 3 and Table S2.

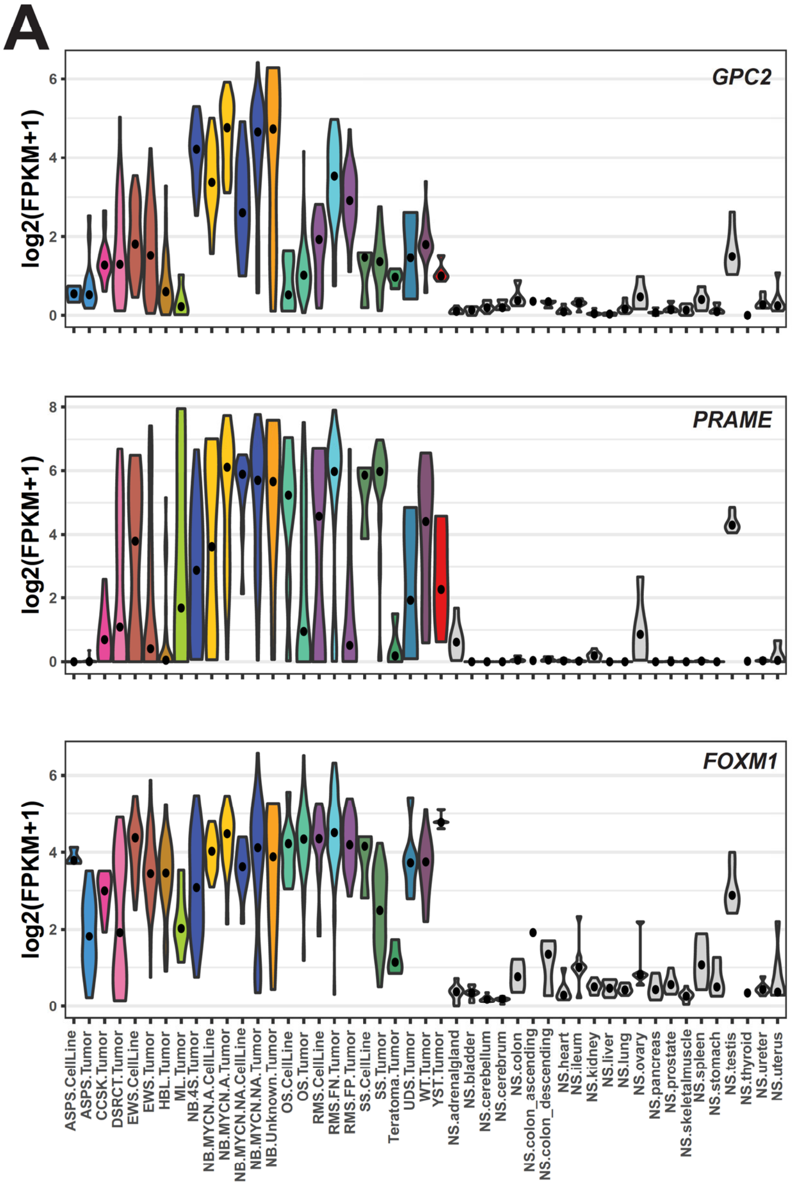

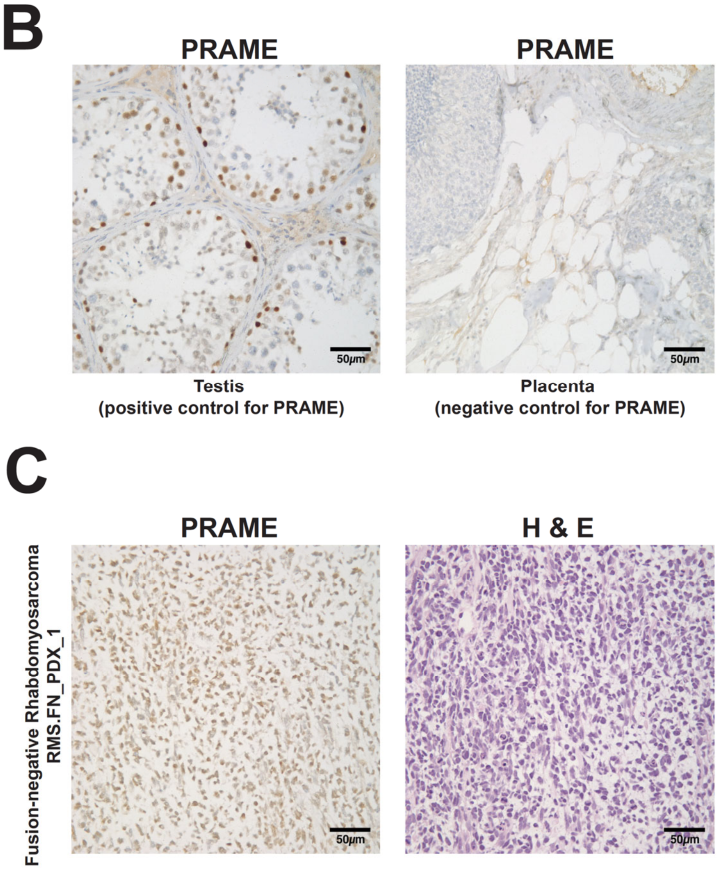

D

Sample ID	EWS_PDX_1	EWS_PDX_2	EWS_PDX_3*	OS_PDX_2	OS_Tumor_1*	RMS.FN_PDX_1	RMS.FN_PDX_2*	RMS.FN_PDX_3	RMS.FP_PDX_1	RMS.FP_PDX_2	RMS.FP_PDX_3	SS_PDX_1
IHC score	-	-	++	+	++	++	++	+	-	-	-	+

Figure S6. Expression of cancer-specific genes. (A) RNA expression of cancer-specific genes, GPC2, FOXM1, and PRAME, in pediatric tumors, cell lines, and normal tissues. **(B)** Positive (testis) and negative control (placenta) for immunohistochemistry (IHC) of PRAME protein. **(C)** IHC of PRAME protein on a fusion-negative RMS. **(D)** PRAME IHC staining intensity scores of 12 patient and patient derived xenograft (PDX) tumors (-, no staining; +, weak staining; ++, strong staining). Related to Figure 4 and Table S3.

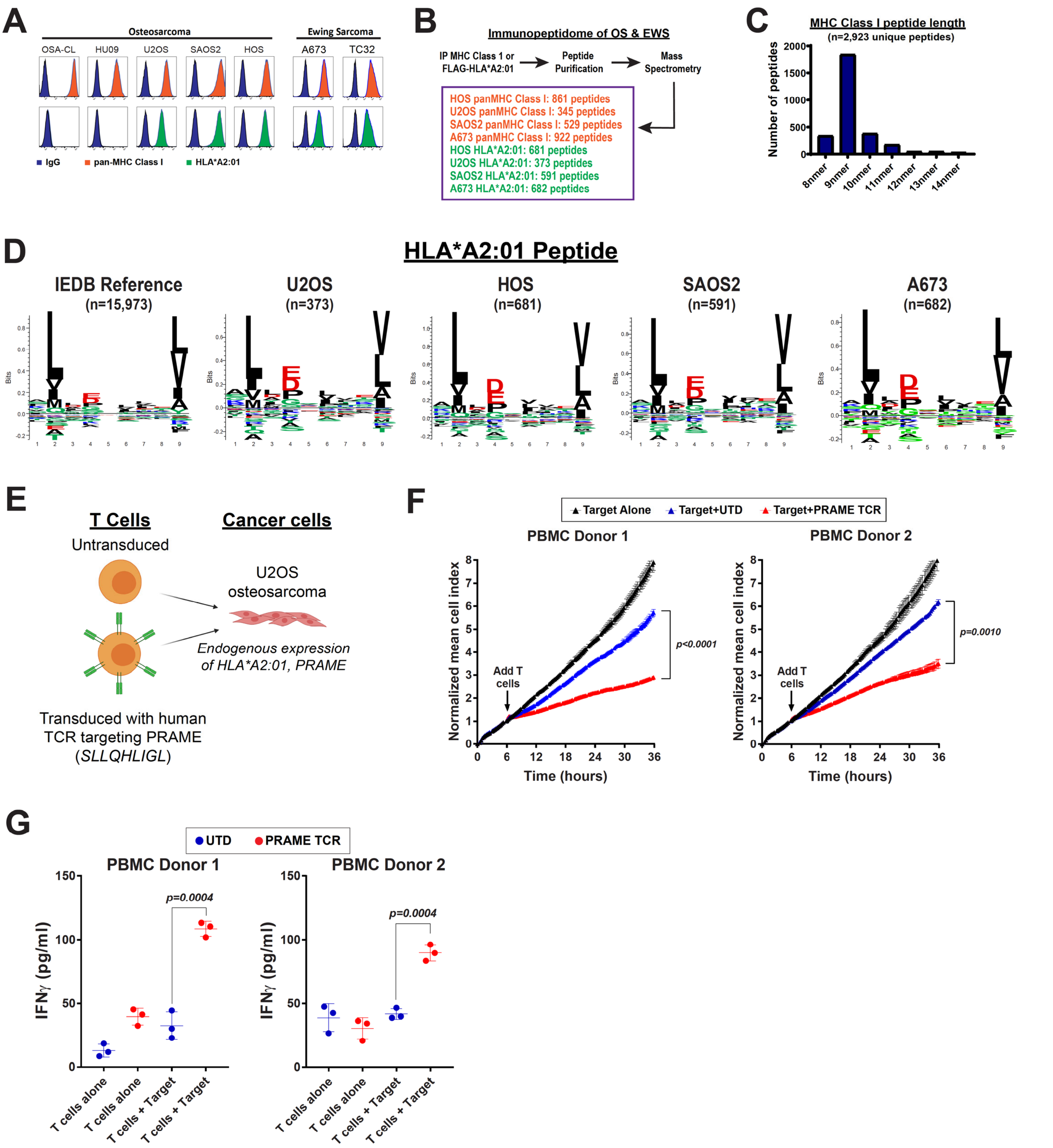

Figure S7. MHC class I immunopeptidome analysis identifies PRAME as an adoptive cell therapy (ACT) target . (A) Flow cytometry of total MHC class I or HLA*A2:01 specific surface expression across human OS and EWS cell lines. **(B)** Workflow for identifying peptides presented by MHC class I in cell lines. **(C)** Size distribution of all unique peptides presented by MHC class I in U2OS, HOS, SAOS2, and A673 cells. **(D)** Using Seq2Logo (Thomsen and Nielsen, 2012), peptide sequences identified by FLAG-HLA*A2 immunopeptidome studies in OS and EWS cells are depicted with a reference logo for HLA*A2:01 presented peptides generated from the IEDB peptide database (Vita et al., 2019). **(E)** Schematic for co-culture of PRAME TCR expressing T cells (from PBMCs of two healthy doners) with U2OS cells, an HLA*A2:01(+) PRAME(+) cell line which we found to present PRAME peptide SLLQHLIGL (Amir et al., 2011). **(F)** TCR transduced or untransduced T cells were co-cultured with U2OS target cells at an E:T ratio of 3:1. Growth data were acquired using a xCELLigence Real-Time Cell Analysis assay and normalized to the time of T cell addition. Data reported as mean ± SEM (n=3). **(G)** MSD cytokine quantification of IFNγ 18 hours after co-culture of T cells with U2OS target cells as in (B). Data reported as mean ± SEM (n=3). Related to Figure 5, Tables 2 & S4.
